# Detection of Pol IV/RDR2-dependent transcripts at the genomic scale in *Arabidopsis* reveals features and regulation of siRNA biogenesis

**DOI:** 10.1101/gr.182238.114

**Published:** 2015-02

**Authors:** Shaofang Li, Lee E. Vandivier, Bin Tu, Lei Gao, So Youn Won, Shengben Li, Binglian Zheng, Brian D. Gregory, Xuemei Chen

**Affiliations:** 1 Department of Botany and Plant Sciences, Institute of Integrative Genome Biology, University of California, Riverside, California 92521, USA;; 2 Department of Biology, University of Pennsylvania, Philadelphia, Pennsylvania 19104, USA;; 3 State Key Laboratory of Genetic Engineering and Institute of Plant Biology, School of Life Sciences, Fudan University, Shanghai 200433, China;; 4 Howard Hughes Medical Institute, University of California, Riverside, California 92521, USA

## Abstract

Twenty-four-nucleotide small interfering (si)RNAs are central players in RNA-directed DNA methylation (RdDM), a process that establishes and maintains DNA methylation at transposable elements to ensure genome stability in plants. The plant-specific RNA polymerase IV (Pol IV) is required for siRNA biogenesis and is believed to transcribe RdDM loci to produce primary transcripts that are converted to double-stranded RNAs (dsRNAs) by RDR2 to serve as siRNA precursors. Yet, no such siRNA precursor transcripts have ever been reported. Here, through genome-wide profiling of RNAs in genotypes that compromise the processing of siRNA precursors, we were able to identify Pol IV/RDR2-dependent transcripts from tens of thousands of loci. We show that Pol IV/RDR2-dependent transcripts correspond to both DNA strands, whereas the RNA polymerase II (Pol II)-dependent transcripts produced upon derepression of the loci are derived primarily from one strand. We also show that Pol IV/RDR2-dependent transcripts have a 5′ monophosphate, lack a poly(A) tail at the 3′ end, and contain no introns; these features distinguish them from Pol II-dependent transcripts. Like Pol II-transcribed genic regions, Pol IV-transcribed regions are flanked by A/T-rich sequences depleted in nucleosomes, which highlights similarities in Pol II- and Pol IV-mediated transcription. Computational analysis of siRNA abundance from various mutants reveals differences in the regulation of siRNA biogenesis at two types of loci that undergo CHH methylation via two different DNA methyltransferases. These findings begin to reveal features of Pol IV/RDR2-mediated transcription at the heart of genome stability in plants.

In plants and mammals, DNA methylation influences gene expression and represses transposable elements (TEs) to ensure genome stability. DNA methylation occurs at CG, CHG, and CHH (H represents A, C, or G) sequence contexts in plants (Law and Jacobsen 2010). In *Arabidopsis*, the methyltransferases DRM2 and CMT2 establish DNA methylation in all sequence contexts and maintain asymmetric CHH methylation (Cao and Jacobsen 2002; Zemach et al. 2013). The maintenance of symmetric CG and CHG methylation is mediated by MET1 and CMT3, respectively (Stroud et al. 2013).

In *Arabidopsis*, RNA-dependent DNA Methylation (RdDM) mediated by DRM2 deposits DNA methylation at TEs to cause their transcriptional silencing (Wierzbicki et al. 2008). Twenty-four-nucleotide (nt) siRNAs serve as the sequence determinants that guide DRM2 to RdDM target loci (Mosher et al. 2008). The plant-specific RNA polymerase IV (Pol IV) is thought to transcribe the RdDM loci to produce single-stranded RNAs (ssRNAs), which are converted to double-stranded RNAs (dsRNAs) by RNA-dependent RNA polymerase 2 (RDR2) (Xie et al. 2004; Jia et al. 2009). DICER-LIKE 3 (DCL3) cleaves the dsRNAs to generate 24-nt siRNAs (Cho et al. 2008; Liu et al. 2009), which associate with AGO4 (Qi et al. 2006). Another plant-specific RNA polymerase, Pol V, produces nascent noncoding transcripts that recruit siRNA-containing AGO4 to RdDM loci (Wierzbicki et al. 2009) with the assistance of both the SUVH2/9 proteins (Johnson et al. 2014) and the DDR complex (composed of DRD1, DMS3, and RDM1) (Zhong et al. 2012; Johnson et al. 2014). This association aids the recruitment of DRM2 leading to cytosine methylation (Law et al. 2010; Zhong et al. 2014). In the genome, loci that produce siRNAs are highly correlated with those that harbor CHH methylation (Lister et al. 2008). Loss of siRNAs in mutants of *NRPD1*, encoding the largest subunit of Pol IV, or *RDR2* results in decreased CHH methylation at numerous loci, usually those residing in euchromatic chromosomal arms and requiring DRM2 for methylation (Wierzbicki et al. 2012). Another pathway mediated by CMT2 together with DDM1 and histone H1 also maintains CHH methylation, but mainly acts at pericentromeric regions (Zemach et al. 2013; Stroud et al. 2014). Together, DRM2 and CMT2 are responsible for nearly all CHH methylation in the genome, and the DRM2-targeted and CMT2-targeted sites are nonoverlapping. Although siRNAs are generated at both DRM2-targeted and CMT2-targeted sites, siRNAs are not required for the maintenance of CHH methylation at CMT2-targeted sites (Zemach et al. 2013; Stroud et al. 2014).

Many factors that participate in siRNA biogenesis are known. Some, such as Pol IV and RDR2, are essential, whereas others, such as DCL3, CLASSY1, and SHH1, play a more limited role (Henderson et al. 2006; Smith et al. 2007; Law et al. 2011, 2013). In the absence of DCL3, which generates 24-nt siRNAs, DCL2 and DCL4 produce endogenous siRNAs of 22 nt and 21 nt, respectively (Allen et al. 2005; Wang et al. 2011). Although Pol IV is purported to produce siRNA precursors, Pol IV-dependent transcripts have never been reported. One difficulty in the detection of Pol IV-dependent transcripts is that they are probably short-lived, because they are likely quickly cleaved by DCL proteins upon their conversion into dsRNAs. The second difficulty lies in the fact that siRNA loci are silenced in wild type and derepressed in Pol IV mutants (Herr et al. 2005; Pontier et al. 2005). This prevents the identification of Pol IV-dependent transcripts by searching for RNAs that are diminished in Pol IV mutants. The lack of knowledge of the Pol IV-dependent transcripts impedes a mechanistic understanding of siRNA biogenesis.

We reasoned that comparing RNAs between *NRPD1* and *nrpd1* genotypes in a *dcl2 dcl3 dcl4* triple mutant background would circumvent the difficulties in detecting Pol IV-dependent transcripts. In the *dcl2 dcl3 dcl4* background, Pol IV-dependent transcripts should be stabilized due to reduced processing by the DCLs. In addition, in the *dcl2 dcl3 dcl4* mutant background, RdDM loci are already derepressed (Xie et al. 2004; Henderson et al. 2006) such that loss of function in *NRPD1* would not cause any further derepression. Therefore, we sought to identify Pol IV-dependent transcripts by RNA sequencing (RNA-seq) and Pol IV/RDR2-dependent transcripts by dsRNA-seq in *dcl2 dcl3 dcl4* and *dcl2 dcl3 dcl4 nrpd1*. This effort led to the identification of Pol IV/RDR2-dependent transcripts from tens of thousands of genomic loci. Further molecular and bioinformatics analyses revealed features of Pol IV/RDR2-dependent transcripts as well as the genetic and epigenetic requirements for Pol IV transcription.

## Results

### Genome-wide discovery of Pol IV-dependent transcripts as siRNA precursors

**Figure 1.**
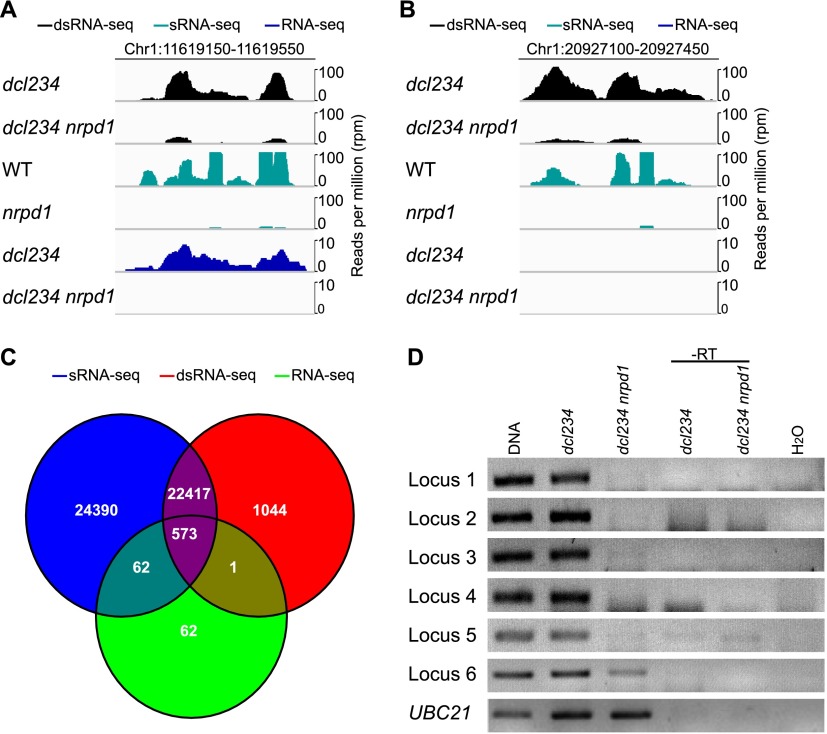
Genome-wide discovery of P4RNAs as P4siRNA precursors. (*A*,*B*) Genome browser views of small RNA reads and P4RNA reads at two representative P4siRNA loci. The read counts (in reads per million [RPM]) include reads from both strands. The *top* two, *middle* two, and *bottom* two rows represent reads from dsRNA-seq, sRNA-seq, and RNA-seq, respectively. In *A*, P4RNAs were detected by both dsRNA-seq and RNA-seq. In *B*, P4RNAs were only detected by dsRNA-seq. (*C*) Venn diagram showing the overlap of P4RNA regions discovered through dsRNA-seq or RNA-seq with P4siRNA regions discovered through sRNA-seq. Note that dsRNA-seq and RNA-seq were conducted with *dcl234* and *dcl234 nrpd1*, and sRNA-seq was conducted with WT and *nrpd1*. (*D*) Random-primed RT-PCR analysis of P4RNAs discovered through RNA-seq on RNA samples from *dcl234* and *dcl234 nrpd1*. Genomic DNA was included as the positive control for the PCR. Reverse transcriptase (-RT) was omitted from the reverse transcription reactions. “-RT” and H_2_O (no RNAs in the reactions) served as negative controls. The genomic locations of the loci can be found in Supplemental Table S1.

To detect Pol IV-dependent transcripts, we compared the transcriptome of the *dcl2-1 dcl3-1 dcl4-2 nrpd1-3* quadruple mutant with that of the *dcl2-1 dcl3-1 dcl4-2* triple mutant (hereafter referred to as *dcl234*) through RNA-seq. Three biological replicates were conducted for each genotype using inflorescences containing unopened flower buds. To derive Pol IV-dependent siRNA loci from the same tissue types, small RNA sequencing (sRNA-seq) was performed with wild-type (WT) and *nrpd1-3* inflorescences. RNA-seq revealed 698 regions showing statistically significant reduction in transcript levels in *dcl234 nrpd1* relative to *dcl234* (Fig. 1A–C; Supplemental Fig. S1). From sRNA-seq, 47,442 Pol IV-dependent siRNA (hereafter referred to as P4siRNA) regions were identified (Fig. 1A–C). Of the 698 regions that generated Pol IV-dependent transcripts (hereafter referred as P4RNAs), 635 overlapped with the P4siRNA regions (Fig. 1C), suggesting that the 635 regions are potential siRNA precursor regions. Twenty-two of these regions (Supplemental Table S1) were randomly selected for detection of P4RNAs by RT-PCR. P4RNAs were detected at all these loci in *dcl234*; these transcripts were either nondetectable or were reduced in abundance in *dcl234 nrpd1* (Fig. 1D; Supplemental Fig. S2A). Therefore, our RNA-seq efforts resulted in the identification of hundreds of regions generating P4RNAs.

The 635 regions shown to produce P4RNAs above only constituted 1.3% of the 47,442 P4siRNA regions. We found that 98% of the P4siRNA regions had little read coverage in the RNA-seq libraries. In *dcl234*, ∼90% of the reads in the RNA-seq libraries were from genic regions, and <5% of the reads were from P4siRNA loci (Supplemental Fig. S2B). Enrichment for P4RNAs in the total RNA population was necessary for the discovery of more P4RNAs.

P4RNAs are thought to be converted to dsRNAs by RDR2 before being processed to P4siRNAs, so the P4RNAs should exist as dsRNAs in the *dcl234* background. We sought to confirm the dsRNA nature of P4RNAs that were detected through RNA-seq above. We performed strand-specific RT-PCR using region- and strand-specific primers for reverse transcription. Indeed, transcripts corresponding to both DNA strands were detected in *dcl234*, and the abundance of transcripts was greatly reduced in *dcl234 nrpd1* (Supplemental Fig. S3). Therefore, P4RNAs could be potentially enriched by separation of dsRNAs from ssRNAs.

We performed three biological replicates of dsRNA-seq in *dcl234* and *dcl234 nrpd1* to enrich for P4RNAs (Zheng et al. 2010). Indeed, the percentage of gene-mapping reads was greatly reduced in dsRNA-seq compared to that from RNA-seq (Supplemental Fig. S2B). Although 35% of the reads mapped to P4siRNA loci in *dcl234*, only 5% did in *dcl234 nrpd1* (Supplemental Fig. S2B), suggesting that there was differential expression at P4siRNA loci between the two genotypes. Indeed, 24,035 regions were found to have a statistically significant reduction in transcript abundance in *dcl234 nrpd1* (Fig. 1A,B; Supplemental Fig. S1); 22,990 of these regions overlapped with the 47,442 P4siRNA regions (Fig. 1C). We consider these 22,990 regions as generating detectable P4siRNA precursors.

Having detected P4RNA-generating regions, we next asked whether all these regions produce P4siRNAs. Our sRNA-seq detected P4siRNAs at 22,990 of the 24,035 regions where P4RNAs were detected by dsRNA-seq. For the 1045 regions from which P4siRNAs were not detected, 946 showed a reduction in small RNA read abundance in *nrpd1* relative to wild type, but these regions did not pass our stringent filter for the definition of differential P4siRNA expression (fourfold reduction in *nrpd1* relative to WT with *P*-value < 0.01). Therefore, these regions were also likely to produce P4siRNAs. This suggests that most (if not all) P4RNAs serve as P4siRNA precursors.

A previous study identified 982 genomic loci bound by Pol IV, among which 787 had detectable siRNA production (Law et al. 2013). The P4RNA-generating regions overlapped with 445 of the 982 regions bound by Pol IV and 405 of the 787 regions producing siRNAs. This does not suggest that only half the Pol IV-occupied regions produce P4RNAs, but rather, this was likely due to the fact that our approach only uncovered P4RNAs at approximately half the regions generating P4siRNAs in the genome (see below).

### RDR2 has a similar effect as Pol IV on the abundance of P4RNAs

**Figure 2.**
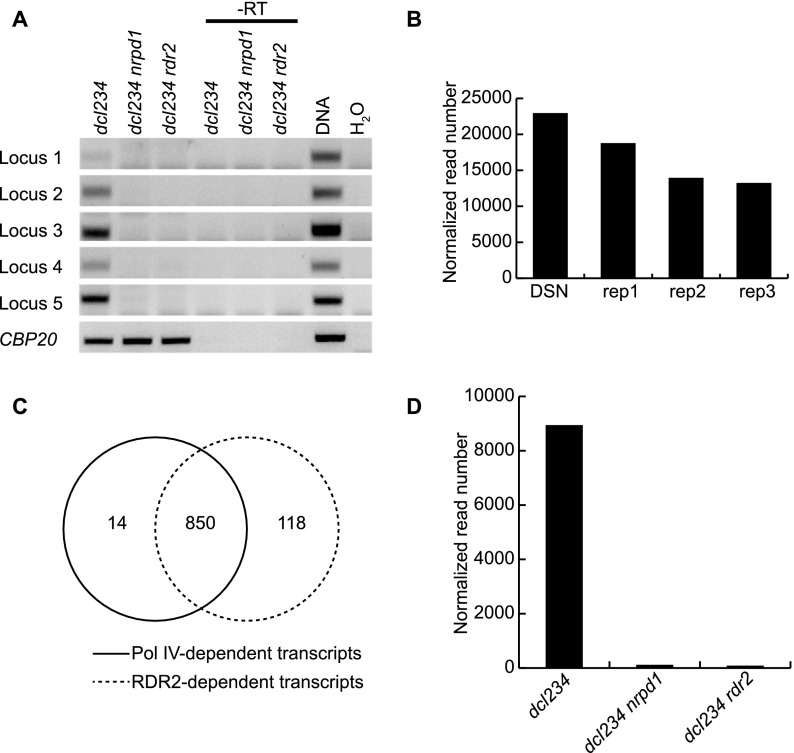
RDR2 has a similar effect as Pol IV on the abundance of P4RNAs. (*A*) Detection of P4RNAs by RT-PCR. Random-primed RT-PCR was performed on *dcl234*, *dcl234 nrpd1*, and *dcl234 rdr2* to detect P4RNAs from five loci (Supplemental Table S1). PCRs with genomic DNA and H_2_O (no RNAs in the reactions) were included as positive and negative controls, respectively. (-RT) Reverse transcription was performed in the absence of reverse transcriptase. *CBP20*, a genic transcript, was included as a loading control. (*B*) DSN normalization moderately enriched the coverage of reads at P4RNA loci by RNA-seq. The total numbers of normalized reads at 47,442 P4siRNA loci from one replicate of *dcl234* RNA-seq-DSN and three replicates of *dcl234* RNA-seq are shown. (*C*) Venn diagram showing the overlap between regions with Pol IV-dependent transcripts and regions with RDR2-dependent transcripts as determined by RNA-seq-DSN of *dcl234*, *dcl234 nrpd1*, and *dcl234 rdr2*. (*D*) Abundance of Pol IV- and RDR2-dependent RNAs at the 850 Pol IV- and RDR2-dependent loci in *C*. The total numbers of normalized reads at these loci in RNA-seq-DSN are shown.

We tested whether *RDR2* is required for the accumulation of P4RNAs. We evaluated the effects of loss of function in *RDR2* on P4RNA levels by performing RT-PCR on *dcl234*, *dcl234 nrpd1*, and *dcl234 rdr2* at five P4siRNA loci. No transcripts were detected in *dcl234 rdr2* or *dcl234 nrpd1* at these loci (Fig. 2A), indicating that the P4RNAs were dependent on both Pol IV and RDR2. The complete lack of P4RNAs in *dcl234 rdr2* was surprising, because we expected to be able to detect P4RNAs in the absence of *RDR2* based on the current RdDM model in which Pol IV generates an ssRNA that is converted to dsRNA by RDR2.

To further examine the in vivo effects of the *rdr2* mutation, we performed RNA-seq with *dcl234*, *dcl234 nrpd1*, and *dcl234 rdr2*. To increase the sensitivity of RNA-seq, we enriched for low abundance transcripts through DSN normalization (see Methods), which resulted in a moderate increase in read coverage at P4siRNA loci (Fig. 2B). As a result, 864 P4RNA regions were identified by comparing *dcl234* to *dcl234 nrpd1* (fourfold difference, *P*-value < 0.01) in RNA-seq-DSN as compared to 698 from RNA-seq (described previously). With the same criteria (fourfold difference, *P*-value < 0.01), 968 regions were found to produce transcripts in *dcl234* relative to *dcl234 rdr2*. Eight hundred fifty regions were common (Fig. 2C), suggesting that the transcripts were dependent on both Pol IV and RDR2. Furthermore, at these 850 loci, the abundance of residual reads from *dcl234 rdr2* was not any higher than that from *dcl234 nrpd1* (Fig. 2D). The results of RT-PCR and RNA-seq-DSN suggest that RDR2 has the same effect on the production of P4RNAs as does Pol IV.

### Assembly of Pol IV/RDR2-dependent transcripts and examination of their surrounding genomic features

With the regions generating P4RNAs known, we next assembled P4RNAs using reads from the dsRNA-seq libraries (see Methods). A total of 17,606 P4RNAs were assembled (Supplemental Dataset 1), with most being in the range of 100–500 nt (Supplemental Fig. S4).

**Figure 3.**
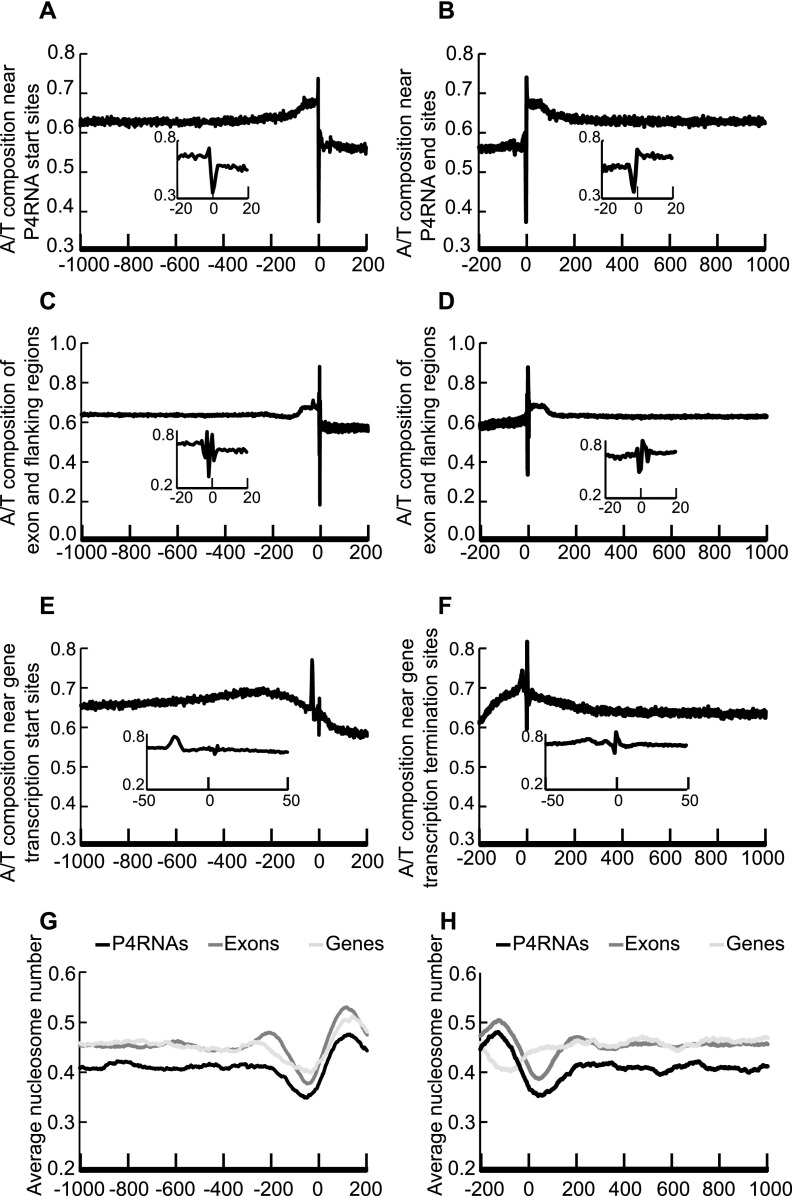
Genomic features of P4RNA and surrounding regions. A/T composition (*A–F*) and nucleosome occupancy (*G–H*) were examined at P4RNAs, exons, and genes. Exons and genes were according to TAIR10 annotation. Position 0 refers to the start site of transcription (for P4RNAs and genes) or the beginning of exons (in *A*, *C*, *E*, *G*) or the end site of transcription/end of exons (in *B*, *D*, *F*, *H*). Nucleotide positions upstream of and downstream from position 0 are represented by negative and positive numbers, respectively. Sequences were aligned at position 0 and the proportion of A/T nucleotides at each position is shown in *A*–*F*. (*A*,*B*) The A/T composition near the P4RNA start sites (*A*) or end sites (*B*). (*C*,*D*) The A/T composition of exons and flanking regions. (*E*,*F*) The A/T composition near protein-coding gene transcription start sites (*E*) or termination sites (*F*). In *A*–*F*, the *insets* display close-up views near position 0. (*G*,*H*) Average nucleosome occupancy near the start sites (*G*) or end sites (*H*) of P4RNAs, exons, and genes. The nucleosome positions were derived from published data (Chodavarapu et al. 2010).

A profound A/T enrichment was found for regions surrounding P4RNAs. We aligned all P4RNAs at their 5′ or 3′ ends and determined the proportion of A/T at each nucleotide position in the 1000 nucleotide window upstream of the 5′ end or downstream from the 3′ end. Since the P4RNAs were double-stranded and the actual orientation of P4RNAs was unknown, the 5′ ends of transcripts were defined as the beginning nucleotides on the Watson strand of the TAIR10 reference sequence. The A/T composition was obviously much lower in the P4RNA bodies than the surrounding regions (Fig. 3A,B), which could simply reflect the GC-richness of P4RNA regions. However, in the ∼50-nt regions flanking P4RNA ends, there was a clear increase in A/T richness relative to the regions further away, suggesting that the immediate flanking regions of P4RNAs are A/T rich. A closer examination of the 5′ or 3′ ends showed that the ends had the lowest A/T composition, whereas the flanking nucleotides had higher A/T composition (Fig. 3A,B, insets). Such patterns of A/T distribution were also found for annotated exons (Fig. 3C,D) and at Pol II transcription start sites (TSS) or termination sites (TTS) (Fig. 3E,F), although the A/T skew at TSS and TTS sites was not as strong.

Nucleosomes, units of chromatin that influence the access of protein factors to the DNA, are known to be enriched on exons and A/T poor regions (Chodavarapu et al. 2010). We determined the nucleosome occupancy at P4RNA regions using published nucleosome sequencing data (Chodavarapu et al. 2010). Nucleosomes were depleted at both the 5′ and 3′ flanking sequences of P4RNAs and enriched at the ends of P4RNAs (Fig. 3G,H). Such nucleosome distribution patterns resembled those on exons and at the TSS of genes (Fig. 3G,H; Chodavarapu et al. 2010; Ammar et al. 2012). These results suggest that the initiation of Pol IV and/or RDR2 transcription occurs in A/T-rich and nucleosome-depleted regions.

The genomic distribution of P4RNAs was also examined. P4RNAs were mainly present at intergenic regions—65% of them overlapped with annotated TEs or repeats, and only 9% of them overlapped with genes (Supplemental Fig. S5A). We performed GO analysis on the set of genes overlapping with P4RNA loci. Intriguingly, the GO term “endomembrane system” was highly enriched for the gene set (Supplemental Table S2). To determine whether this unexpected association was due to the concentration of “endomembrane system” genes at pericentromeric regions, we examined the chromosomal distributions of the set of genes overlapping with P4RNAs. We found that the gene set resembled the set of all annotated genes in that the genes were dispersed at euchromatic regions and depleted at pericentromeric regions (Supplemental Fig. S6A).

We next examined the association between regions generating P4RNAs and heterochromatic marks. We first examined the relationship among P4RNAs, P4siRNAs, and CHH regions dependent on DRM2 or CMT2. DRM2- and CMT2-dependent CHH methylation regions were defined as the CHH differentially methylated regions (CHH DMRs) with reduced methylation in *drm1 drm2* and *cmt2* relative to WT, respectively, in a published methylome study (Stroud et al. 2013). Similarly, Pol IV-dependent CHH regions were defined as CHH DMRs between WT and *nrpd1* in the same study (Stroud et al. 2013). Although both DRM2- and CMT2-targeted sites strongly overlapped with regions producing P4siRNAs (Supplemental Fig. S5B), the sites targeted by the two methyltransferases are largely nonoverlapping (Supplemental Fig. S5C; Zemach et al. 2013). Pol IV-dependent CHH regions are mainly targeted by DRM2 (Supplemental Fig. S5C), and the number of Pol IV/DRM2-dependent CHH regions is only half the number of CMT2-dependent CHH regions. Therefore, loss of P4siRNAs only leads to reduction in CHH methylation at a small proportion of P4siRNA loci, and these loci are distributed along euchromatic chromosomal arms (Supplemental Fig. S6B; Wierzbicki et al. 2012). We found that the chromosomal distribution of P4RNAs strongly resembled those of total CHH methylation and CMT2-dependent CHH methylation, which peak at pericentromeric regions (Supplemental Fig. S6B; Lister et al. 2008; Zemach et al. 2013). This suggests that loci with detected P4RNAs are largely contributed by those whose CHH methylation is targeted by CMT2, which will be further examined later.

Besides siRNAs and DNA methylation, H3 lysine 27 monomethylation (H3K27me1) and H3 lysine 9 dimethylation (H3K9me2) are two other common heterochromatic marks, for which the genomic distributions were profiled through ChIP-chip (Roudier et al. 2011; Deleris et al. 2012). We found that these two marks exhibited similar chromosomal distributions as P4RNAs—all were enriched at pericentromeric regions (Supplemental Fig. S6C).

### Features of Pol IV/RDR2-dependent transcripts

**Figure 4.**
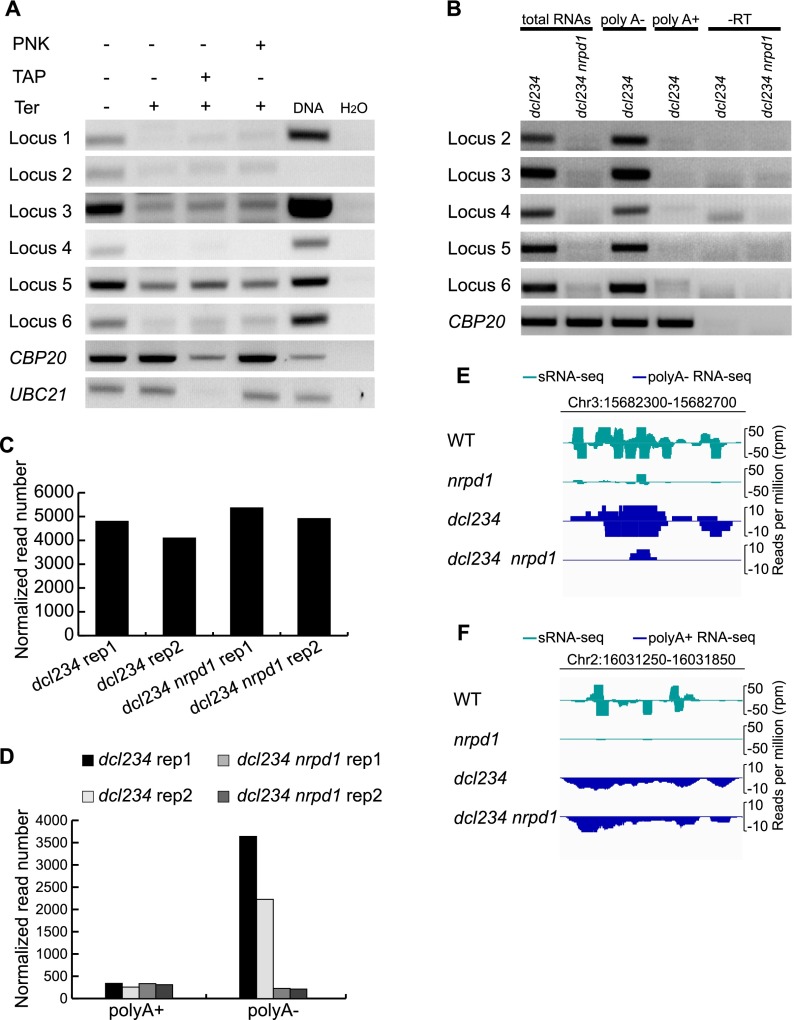
Features of P4RNAs. (*A*) Determination of the 5′ end structure of P4RNAs. Total RNAs from *dcl234* were treated (+) or not (−) with various enzymes and subjected to random-primed RT-PCR to detect specific P4RNAs (loci 1–6) (Supplemental Table S1) with P4RNA-specific primers. (PNK) Polynucleotide kinase; (TAP) tobacco acid pyrophosphatase; (Ter) Terminator exonuclease. PCRs with genomic DNA and H_2_O (no RNAs in the reactions) were included as positive and negative controls, respectively. Transcripts from two genes, *CBP20* and *UBC21*, were also detected by RT-PCR as controls. As expected, the levels of these RNAs were only reduced by digestion with both TAP and Ter. (*B*) Determination of the 3′ end structure of P4RNAs. Random-primed RT-PCR was performed on total RNAs from *dcl234* and *dcl234 nrpd1*, and poly(A)-enriched and poly(A)-depleted RNAs from *dcl234* to detect specific P4RNAs. (-RT) Reverse transcription was performed in the absence of reverse transcriptase. *CBP20* served as a positive control for poly(A)^+^ RNAs. The *CBP20* RT-PCR products in the poly(A)^−^ fraction probably reflected degradation intermediates. (*C*) Abundance of reads at 1639 P4RNA regions with detectable transcripts in poly(A)^+^ RNA-seq. Two replicates of RNA-seq were conducted and the sum of the numbers of normalized reads is shown. (*D*) Abundance of transcripts at 698 P4RNA regions discovered through the initial RNA-seq, as determined by RNA-seq from poly(A)^+^ and poly(A)^−^ RNAs. The reduction in transcript abundance in *dcl234 nrpd1* was only observed in poly(A)^−^ RNAs, indicating that P4RNAs lack poly(A) tails. (*E*) A genome browser view of reads at a P4siRNA locus on Chromosome 3 from sRNA-seq and poly(A)^−^ RNA-seq. Read abundance is shown for both the Watson (*top*) and Crick (*bottom*) strands. (*F*) A genome browser view of reads at a P4siRNA locus on Chromosome 2 from sRNA-seq and poly(A)^+^ RNA-seq. Read abundance is shown for both the Watson (*top*) and Crick (*bottom*) strands.

The 5′ initiating nucleotides of Pol I and Pol III transcripts have triphosphate groups, and those of Pol II transcripts contain 7-methylguanosine caps. To determine the 5′ end structure of P4RNAs, we performed enzymatic treatments of total RNAs followed by the detection of P4RNAs by RT-PCR. First, we treated total RNAs with no enzyme (control), tobacco acid pyrophosphatase (TAP), which converts 5′ triphosphate or 5′ 7-methylguanylate cap to 5′ monophosphate, or T4 polynucleotide kinase (PNK), which adds a 5′ phosphate group to 5′ hydroxyl RNAs. Next, we digested the RNAs with Terminator, a 5′ to 3′ exonuclease that acts on RNAs with a 5′ monophosphate. Finally, RT-PCR was conducted on these treated RNA samples to detect various P4RNAs. The RNAs treated with Terminator alone showed a dramatic reduction in the abundance of P4RNAs (Fig. 4A), suggesting that a large portion of P4RNAs had a 5′ monophosphate. The samples treated with TAP or PNK followed by Terminator showed similar levels of P4RNAs to the sample treated with Terminator alone (Fig. 4A). The fact that TAP or PNK treatment did not increase the amount of 5′ monophosphate RNAs indicated that P4RNAs primarily had a 5′ monophosphate.

Introns are a common feature of Pol II-dependent transcripts. To determine whether the P4RNAs have introns, we first analyzed reads from *dcl234* dsRNA-seq libraries with TopHat2 (Kim et al. 2013), a widely used software to discover splice junctions for canonical introns. Through TopHat2, 20,521 spliced junctions were reported, with 20,378 junctions being at genic regions and only 59 junctions being at P4RNA regions. As P4RNAs do not necessarily use splice junctions characteristic of Pol II-dependent transcripts, we also used a naïve method that reports all spliced reads, i.e., reads whose 5′ and 3′ portions represent nearby genomic sequences separated by a segment (see Methods). This method predicted 16,018 spliced reads, with 12,670 being at genic regions and only 112 being at P4RNA regions. The potential spliced junctions predicted by the two methods at P4RNA regions were further examined to determine whether they represented true spliced junctions. The levels of transcripts at intron regions should be much lower than those at exon regions. When subjected to the filter that the coverage of “intron” regions is at least five times lower than that of the flanking regions, none of the predicted junctions was retained. This suggests that P4RNAs do not possess introns.

Polyadenylation is part of the maturation process of Pol II-dependent transcripts. To determine whether P4RNAs have poly(A) tails, total RNAs were separated into poly(A)^+^ and poly(A)^−^ fractions followed by the detection of P4RNAs by RT-PCR. P4RNAs were detectable from total RNAs and poly(A)^−^ RNAs, but not from poly(A)^+^ RNAs, suggesting that P4RNAs do not have poly(A) tails (Fig. 4B).

Given that P4RNAs lack poly(A) tails and Pol II-dependent transcripts are expected to have poly(A) tails, we sought to distinguish the two types of transcripts at RdDM loci through the presence or absence of poly(A) tails. Poly(A)^−^ and poly(A)^+^ RNAs were first isolated from two biological replicates of *dcl234* and *dcl234 nrpd1* and subjected to an RNA-seq library construction procedure that preserved the strandedness of the transcripts. In poly(A)^+^ libraries, a total of 1639 P4siRNA loci were found to have read coverage >1 reads per million (RPM), indicating that they were expressed. Transcript abundance at these loci was similar in *dcl234 nrpd1* and *dcl234* (Fig. 4C), suggesting that the poly(A)^+^ transcripts were made by Pol II rather than Pol IV. Next we examined the read coverage at the 698 Pol IV-dependent regions discovered through the initial RNA-seq experiment (reported at the beginning of Results) in the poly(A)^−^ RNA-seq libraries. At 98% of the regions where expression was detected from *dcl234*, decreased expression in *dcl234 nrpd1* was also observed (Fig. 4D). In addition, the expression of these 698 regions as determined by poly(A)^+^ RNA-seq was very low, and decreased expression in *dcl234 nrpd1* was not observed (Fig. 4D). This confirmed that P4RNAs are present in the poly(A)^−^ RNA fraction and absent from the poly(A)^+^ RNA fraction.

Our previous studies showed that a partial loss-of-function mutation in a Pol II subunit gene compromised P4siRNA biogenesis at some RdDM loci. This raised the question of whether Pol II-dependent transcripts at RdDM loci are directly channeled to P4siRNA biogenesis or Pol II promotes P4siRNA biogenesis indirectly, such as by recruiting Pol IV (Zheng et al. 2009). The ability to distinguish Pol II-dependent transcripts and P4RNAs at RdDM loci allowed us to address this question. If Pol II-dependent transcripts were channeled to P4siRNA production, we would expect to detect dsRNAs from Pol II-dependent transcripts in *dcl234*. At P4siRNA loci, P4RNAs in poly(A)^−^ RNA-seq were derived from two strands as expected (Fig. 4E; Supplemental Fig. S7A). However, transcripts in poly(A)^+^ RNA-seq, presumably Pol II-dependent transcripts, appeared to be mainly derived from one strand (Fig. 4F; Supplemental Figs. S7B, S8). We calculated the ratio of reads from the two strands in poly(A)^+^ RNA-seq at 1639 P4siRNA loci, where Pol II transcription was detectable. Approximately 99% of poly(A)^+^ RNAs at these loci had a ratio of 9:1 or larger between reads derived from the two strands (Supplemental Fig. S7C). This suggests that Pol II-transcribed RNAs were not converted to dsRNAs. We next examined the strand distribution of P4siRNAs at these loci. P4siRNAs were present at some of the loci in *dcl234*, probably because DCL1 was able to produce P4siRNAs. The reads for P4siRNAs were derived from two strands, whereas the Pol II-transcribed poly(A)^+^ RNAs were from one strand (Supplemental Fig. S8). In *dcl234 nrpd1*, the P4siRNAs were depleted, suggesting that the P4siRNAs were derived from Pol IV. The fact that Pol II-dependent RNAs from loci that generate P4siRNAs are only from one strand (Supplemental Fig. S8) and that no P4siRNAs are present in *dcl234 nrpd1* suggests that Pol II-dependent transcripts are not channeled to P4siRNA production.

### The decreased CHH DNA methylation in *dcl234* is correlated to compromised Pol IV transcription

Our dsRNA-seq effort uncovered 22,990 regions producing P4RNAs, less than half the 47,442 regions that produce P4siRNAs. Thus, we interrogated why P4RNAs were not detected from half the P4siRNA-generating loci. We examined the 24,452 P4siRNA regions from which P4RNAs were not detected and found that 72% of the regions had low read coverage of <0.9 RPM in both *dcl234* and *dcl234 nrpd1* dsRNA-seq libraries, which made it impossible to make any comparisons between the two genotypes (Supplemental Fig. S9A). Therefore, low levels of the P4RNAs were the major reason prohibiting their discovery.

**Figure 5.**
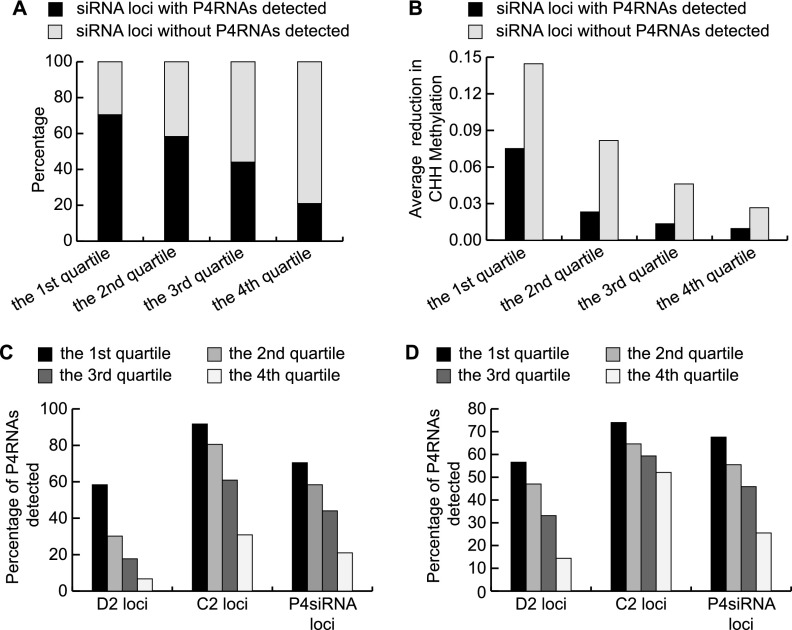
Decreased CHH DNA methylation in *dcl234* compromises Pol IV transcription. (*A*–*C*) P4siRNA loci are divided into four quartiles according to P4siRNA abundance in WT, with the first quartile containing loci with the highest levels of P4siRNAs. (*A*) The percentage of P4siRNA loci with and without P4RNAs detected in our dsRNA-seq for the four quartiles. (*B*) The levels of CHH methylation decrease in *dcl234* compared to WT for the four quartiles of P4siRNA loci with and without precursors detected. The decrease in CHH methylation was calculated using published methylome data (Stroud et al. 2013). (*C*) The percentage of P4RNAs detected at D2 and C2 siRNA loci for the four quartiles. Included in the analysis were 13,479 D2; 19,039 C2; and 47,742 total P4siRNA loci. (*D*) Correlation between P4RNA discovery and levels of CHH methylation at the siRNA loci. D2 and C2 siRNA loci are divided into four quartiles according to their CHH methylation levels in *dcl234*. The percentage of loci with P4RNAs detected in each quartile is shown. As CHH methylation levels decrease, the success rate of P4RNA discovery also decreases in total P4siRNA loci. In terms of P4RNA discovery, D2 loci are more sensitive to levels of CHH methylation.

We next asked whether the low levels of P4RNAs at these regions in *dcl234* were attributable to the fact that these regions have low Pol IV activity in WT. The output of Pol IV activity is P4siRNAs. We divided all regions producing P4siRNAs in WT into four quartiles according to the abundance of P4siRNAs. The percentage of P4RNAs discovered was calculated for each quartile. As expected, with the decrease in P4siRNA abundance, the percentage of P4RNAs discovered also decreased. However, even in the first quartile that contained regions with the most abundant P4siRNAs in WT, still 30% of the regions lacked detectable P4RNAs in *dcl234* (Fig. 5A). Therefore, our approaches failed to detect P4RNAs at some of the loci that generate abundant P4siRNAs and are thus predicted to also generate high levels of P4RNAs.

We next examined whether levels of P4RNAs in *dcl234* were correlated to levels of CHH DNA methylation. The CHH DNA methylation levels were examined separately for P4siRNA loci with or without P4RNAs detected. The average CHH DNA methylation levels at the two types of loci were similar in WT (Supplemental Fig. S9C), but different in *dcl234*; the type without P4RNAs detected had much lower levels of CHH methylation than the type with P4RNAs detected (Fig. 5B; Supplemental Fig. S9D). Therefore, it appeared that CHH methylation correlated with the production of P4RNAs.

We examined whether P4RNAs were affected differently by CHH methylation at DRM2- and CMT2-targeted sites, which will be referred to as D2 and C2 loci for simplicity. The P4siRNAs produced from these two types of loci will be referred to as D2 and C2 siRNAs. First, the relative abundance of D2 and C2 siRNAs was determined by sRNA-seq in WT. Although the number of C2 loci was larger than that of D2 loci, the total small RNA read number of C2 siRNAs was much smaller than that of D2 siRNAs even when total P4siRNAs or only 21-nt, 22-nt, 23-nt, or 24-nt P4siRNAs were separately considered (Supplemental Fig. S9B). Next, we calculated the percentage of P4RNA discovery in *dcl234* at D2 and C2 loci separately. Although the average abundance of D2 siRNAs was higher than C2 siRNAs, P4RNAs were detected at 38% of D2 loci versus 62% of C2 loci. The difference was even more obvious when D2 and C2 loci belonging to the lowest quartile of P4siRNA abundance were considered (Fig. 5C). We observed a strong correlation between P4RNA discovery and CHH DNA methylation at D2 loci. When D2 sites were divided into four quartiles according to their CHH DNA methylation levels in *dcl234*, the percentage of D2 P4RNA discovery decreased with decreasing CHH DNA methylation (Fig. 5D). Similarly, the abundance of P4RNAs at D2 sites, as revealed by dsRNA-seq in *dcl234*, also decreased with decreasing CHH DNA methylation (Supplemental Fig. S10A). These trends were not found for C2 sites (Fig. 5D; Supplemental Fig. S10A). The correlation between P4siRNA abundance and levels of CHH methylation was also examined in *dcl234* (Supplemental Fig. S10B,C) and WT (Supplemental Fig. S10D,E). The abundance of D2 siRNAs but not C2 siRNAs decreased with decreasing CHH DNA methylation. In summary, Pol IV transcription appeared to depend on CHH DNA methylation to a greater extent at D2 sites than at C2 sites.

### Genetic requirements for P4siRNA biogenesis

Previous studies demonstrated that Pol IV, RDR2, and DCL3 are responsible for the biogenesis of P4siRNAs and that CHH DNA methylation and H3K9me2 affect P4siRNA accumulation. By utilizing published sRNA-seq, ChIP-seq, and methylome data (Supplemental Table S3; Roudier et al. 2011; Deleris et al. 2012; Lee et al. 2012; Law et al. 2013; Stroud et al. 2013, 2014), we further explored the genetic requirements for P4siRNA production.

**Figure 6.**
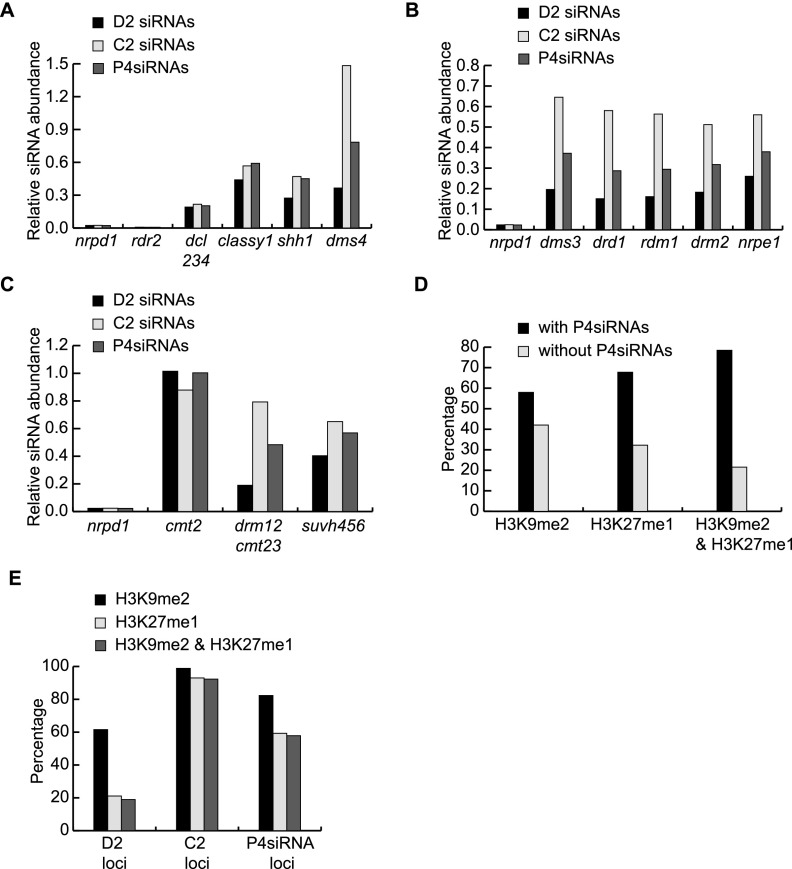
RdDM genes, epigenetic marks, and P4siRNA biogenesis. (*A*–*C*) Effects of mutations in various CHH methylation pathway genes on P4siRNA biogenesis. The relative abundance of D2, C2, and total P4siRNAs in various mutants compared to WT is shown. The analysis was performed with published sRNA-seq data (Lee et al. 2012; Law et al. 2013; Stroud et al. 2014). For each genotype, reads corresponding to P4siRNA loci were normalized against small RNAs from non-P4siRNA loci. P4siRNA loci were defined as those showing differentially expressed siRNAs between WT and *nrpd1* (see Methods). A total of 47,742 total P4siRNA loci, 13,479 D2, and 19,039 C2 loci were used in the analysis. (*A*) Relative siRNA abundance in mutants in genes known to act in P4siRNA biogenesis. (*B*) Relative siRNA abundance in mutants in genes known to act downstream from P4siRNAs in RdDM. (*C*) Relative siRNA abundance in mutants in genes that confer CHH DNA methylation or histone H3K9 methylation. *nrpd1* is included in *B* and *C* for comparison. (*D*,*E*) Overlap between P4siRNA loci and the epigenetic marks H3K9me2 or H3K27me1. Published ChIP-chip data were used to define regions with H3K9me2 or H3K27me1 (Roudier et al. 2011; Deleris et al. 2012). (*D*) Regions with H3K9me2, H3K27me1, or both H3K9me2 and H3K27me1 were divided into 500-bp windows. The number of windows where P4siRNAs were present or not was counted, and the percentage of total windows is shown. (*E*) The percentage of P4siRNA loci with H3K9me2, H3K27me1, or both. The number of D2, C2, and total P4siRNA loci with H3K9me2, H3K27me1, or both marks was determined, and the percentage of these total loci is shown.

The levels of P4siRNAs were first examined in WT and mutants in genes participating in P4siRNA biogenesis such as *DCL3*, *RDR2*, *NRPD1*, *SSH1*, *CLSY1*, and *DMS4*. D2, C2, and total P4siRNAs were equally affected in *dcl234*, *rdr2*, and *nrpd1* (Fig. 6A). In *clsy1*, *ssh1*, and *dms4*, D2 siRNA levels were similarly decreased but not completely eliminated, and the reduction in P4siRNA abundance correlated with a reduction in CHH methylation in the three genotypes (Fig. 6A; Supplemental Fig. S11A). At C2 loci, P4siRNA levels were decreased in *clsy1* and *ssh1* but increased in *dms4*, and these changes in P4siRNA levels were not accompanied by appreciable changes in CHH methylation (Fig. 6A; Supplemental Fig. S11B). Therefore, a correlation between P4siRNA accumulation and CHH methylation is only true for D2 loci. Another conclusion is that all these genes, with the exception of *DMS4*, are required for P4siRNA biogenesis at both D2 and C2 loci.

The levels of P4siRNAs were also examined in mutants of genes participating in the RdDM pathway downstream from P4siRNA biogenesis, such as *DMS3*, *DRD1*, *RDM1*, *DRM2*, and *NRPE1*. Mutations in these genes all resulted in a near elimination of CHH methylation at D2 loci (Supplemental Fig. S11A) but had almost no effect on CHH methylation at C2 loci (Supplemental Fig. S11B). P4siRNA levels were also reduced in these mutants at both D2 and C2 loci, but D2 loci were affected to a greater extent; the remaining P4siRNAs were at 20% and 60% of wild-type levels for D2 and C2 loci, respectively (Fig. 6B). These results were also consistent with a correlation between P4siRNA biogenesis and CHH methylation at D2 loci.

The levels of P4siRNAs were also examined in mutants of genes that confer DNA methylation, such as *DRM2*, *CMT3*, and *CMT2*, or H3K9me2 deposition, such as *SUVH4*, *5*, and *6*. In the *cmt2* mutant, in which CHH methylation is nearly eliminated at C2 loci but unaffected at D2 loci (Supplemental Fig. S11A,B), P4siRNA accumulation was not affected at D2 loci or C2 loci (Fig. 6C). In *drm1 drm2 cmt2 cmt3* (*drm12cmt23*), in which all non-CG methylation is lost and H3K9me2 cannot be maintained because of the loss of non-CG methylation (Supplemental Fig. S11A,B; Stroud et al. 2014), D2 siRNA levels were severely reduced, but C2 siRNAs were only weakly affected (Fig. 6C). In *suvh456*, in which H3K9me2 is lost (Stroud et al. 2014) and CHH methylation at both D2 and C2 loci is partially reduced (Supplemental Fig. S11A,B), D2 and C2 siRNAs were at 40% and 65% of the levels in WT, respectively (Fig. 6C).

The above observations support a tight correlation between CHH methylation and P4siRNA abundance at D2 loci but only a weak correlation at C2 loci. To explore possible contributors to P4siRNA biogenesis at C2 loci, we examined the overlap between P4siRNA loci and repressive epigenetic marks H3K9me2 and H3K27me1 (Roudier et al. 2011; Deleris et al. 2012). P4siRNAs were found at 57% of H3K9me2 regions, 67% of H3K27me1 regions, and 75% of the regions harboring both H3K9me2 and H3K27me1, which may suggest that H3K27me1 and H3K9me2 work together in promoting P4siRNA biogenesis (Fig. 6D). When D2 and C2 loci were separately examined for their overlap with H3K9me2 and H3K27me1, both marks were present at 92% of C2 loci but only 19% of D2 loci (Fig. 6E). This is consistent with prior knowledge that D2 loci are primarily on euchromatic arms, whereas C2 loci are in pericentromeric heterochromatin (Zemach et al. 2013).

**Figure 7.**
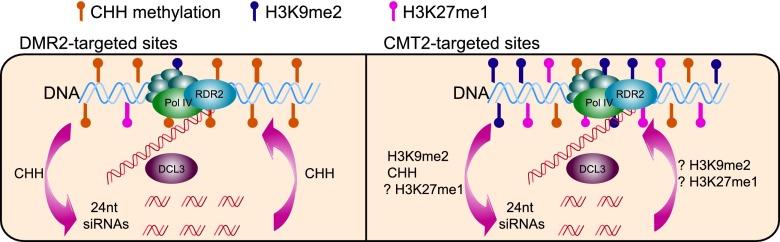
Models on the feedback regulation between Pol IV transcription and epigenetic marks at D2 and C2 loci. At both D2 and C2 loci, P4siRNA biogenesis requires Pol IV, RDR2, and DCL3. At D2 loci with high levels of methylated CHH and relatively low levels of H3K9me2 or H3K27me1, P4siRNAs and CHH methylation are in a tight feedback loop in which P4siRNAs guide CHH methylation, and CHH methylation in turn promotes siRNA biogenesis, probably by recruiting Pol IV. At C2 loci with relatively low levels of methylated CHH and extensive overlap with H3K9me2 or H3K27me1, P4siRNA biogenesis is only moderately affected by the absence of CHH methylation (in *drm12cmt23* and *cmt2*) or H3K9me2 (in *suvh456*). The high percentage of C2 siRNA loci containing both H3K9me2 and H3K27me1 suggests that both epigenetic marks may contribute to Pol IV recruitment at C2 loci.

In summary, D2 and C2 siRNAs share a common biogenesis pathway involving Pol IV, RDR2, and DCL3, but Pol IV transcription at these loci is probably regulated differently by different epigenetic marks (Fig. 7). Compared to C2 siRNAs, D2 siRNAs are highly abundant and are found at euchromatic regions harboring high levels of CHH methylation but low levels of H3K9me2 or H3K27me1 (Supplemental Fig. S11C–F). D2 siRNAs and CHH methylation appear to be under tight feedback regulation—D2 siRNAs are required for the maintenance of CHH methylation, and their biogenesis (probably at the level of Pol IV transcription) is promoted by CHH methylation. In contrast, C2 siRNAs are less abundant and are found at heterochromatic regions with high levels of repressive marks such as H3K9me2 or H3K27me1 (Supplemental Fig. S11C–F). C2 siRNAs are not required to maintain CHH methylation, and their biogenesis is less affected by the loss of CHH methylation, probably because H3K9me2 and H3K27me1 contribute to Pol IV transcription at these loci.

## Discussion

Pol IV is thought to generate the precursors to endogenous siRNAs, which are central players in RdDM in plants. However, Pol IV-derived transcripts have not been detected before, probably owing to their short-lived nature and the transcription of RdDM loci by Pol II in a Pol IV loss of function mutant. In this study, we devised a strategy that enabled the detection of tens of thousands of P4siRNA precursors that we refer to as P4RNAs. The analysis of these P4RNAs provided the following insights into P4siRNA biogenesis.

Specifically, key tenets of the current model of P4siRNA biogenesis have been confirmed. We showed for the first time that Pol IV indeed generates long noncoding RNAs, consistent with the presumed role of Pol IV in transcribing RdDM loci in the current model. Previously, failure to detect Pol IV transcription by a nuclear run-on assay led to the hypothesis that Pol IV in maize is likely a dysfunctional polymerase (Erhard et al. 2009). Our findings are in favor of *Arabidopsis* Pol IV, and maize Pol IV by inference, as a functional polymerase. The fact that long noncoding P4RNAs are from both DNA strands and are absent in an *rdr2* mutant is consistent with the model that P4siRNA precursors are generated by the concerted actions of Pol IV and RDR2. Our findings may also prompt a reconsideration of the current model. Previous biochemical studies show that RDR2 and Pol IV are in the same complex and, in vitro, RDR2 activity requires Pol IV but Pol IV activity does not require RDR2 (Haag et al. 2012). Based on these observations, the current model is that Pol IV transcribes P4siRNA loci and RDR2 converts nascent P4RNAs into dsRNAs (Matzke and Mosher 2014). Our findings not only agree with the notion that Pol IV and RDR2 act together, but also implicate an essential role of RDR2 for Pol IV transcription. If Pol IV activity does not require RDR2 in vivo, we expect to detect P4RNAs in *dcl234 rdr2*. However, detection of P4RNAs either by RT-PCR at specific loci or by RNA-seq at the genomic scale showed that *nrpd1* and *rdr2* mutations were equally defective in the production of these transcripts. This suggests that RDR2 may be required for the recruitment of Pol IV to P4siRNA loci, the transcription activity of Pol IV, or the stability of P4RNAs in vivo.

Our findings also provide new insights into RdDM. We show that P4RNAs are nonpolyadenylated and lack introns, and thus are different from Pol II-dependent transcripts. Using the presence or absence of poly(A) as the distinguishing feature, we found that P4RNAs are derived from both DNA strands, whereas the derepression of RdDM loci results in Pol II transcription from a single strand. The single-stranded nature of Pol II-dependent transcripts from RdDM loci in *dcl234* also suggests that Pol II transcripts are not converted to dsRNAs for P4siRNA production. However, our previous studies revealed a reduction in P4siRNA levels from some RdDM loci in a partial loss-of-function Pol II mutant (Zheng et al. 2009). Together, these data imply that Pol II does not contribute to P4siRNA biogenesis by supplying P4siRNA precursors. Instead, Pol IV recruitment to chromatin was compromised in the Pol II mutant (Zheng et al. 2009), suggesting that Pol II transcription might act to recruit Pol IV. However, we note that this study only examined loci that are already under surveillance by RdDM. We cannot rule out that Pol II-derived transcripts may be used directly in siRNA production when a naïve element is first introduced into a genome.

The lack of introns in P4RNAs also has implications. Several splicing related proteins were reported to affect both P4siRNA abundance and CHH methylation, although their effects are less prominent than that of Pol IV (Zhang et al. 2013). The absence of introns in P4RNAs suggests that these splicing factors promote P4siRNA biogenesis either indirectly through their splicing functions on genes or directly through splicing-independent functions on P4RNAs.

A surprising finding was that the 5′ ends of P4RNAs bear a monophosphate. The 5′ end of a primary transcript is expected to bear a 5′ triphosphate, or a cap as in Pol II-derived transcripts. It is possible that the P4RNAs that we detected represent processed transcripts. Alternatively, Pol IV or RDR2 may use 5′ monophosphate-containing RNAs as primers to initiate transcription. Regardless, the predominant form of P4RNAs in vivo is the form with a 5′ monophosphate. In this respect, P4RNAs resemble rRNAs, which are present in vivo as processed forms with a 5′ monophosphate (Dahlber et al. 1978; Unfried and Gruendler 1990). It is of note that the P4RNAs are also products of RDR2; therefore, the features of the 5′ and 3′ ends reflect co- or post-transcriptional events of Pol IV/RDR2 transcription.

A striking finding was the higher A/T composition and lower nucleosome occupancy of the flanking sequences of P4RNAs. This raises the possibility that high A/T composition and absence of nucleosomes promote the initiation and termination of Pol IV transcription. Nucleosome depletion in the 5′ flanking region is immediately followed by nucleosome enrichment 3′ to the transcription start site for P4RNAs. Such a pattern of nucleosome distribution is also found around the transcription start sites of protein-coding genes in diverse eukaryotes (Ammar et al. 2012), and thus represents a common feature of transcription initiation sites for Pol II and Pol IV.

CHH DNA methylation and H3K9me2 are repressive marks in the suppression of transposon expression and both are thought to promote P4siRNA biogenesis. Recent studies have uncovered two parallel pathways of CHH methylation maintenance requiring two different DNA methyltransferases, DRM2 and CMT2. For the DRM2-targeted (D2) sites that are more dispersed within chromosomal arms, P4siRNAs and CHH methylation levels are high, and loss of CHH methylation impedes Pol IV transcription to result in reduced P4siRNA abundance. Therefore, CHH methylation and P4siRNA biogenesis are engaged in a positive feedback loop at D2 sites (Fig. 7). CMT2-targeted (C2) sites are concentrated at pericentromeric regions, where other repressive marks, such as H3K9me2 and H3K27me1, are prevalent (Roudier et al. 2011). At these sites, loss of CHH methylation has a minimal effect on Pol IV transcription as compared to D2 sites, and little impact on P4siRNA abundance (Fig. 7). Although it was found that H3K9me2 promotes P4siRNA accumulation at C2 sites (Stroud et al. 2014; this study), C2 siRNAs are only moderately affected in the *suvh456* mutant that lacks H3K9me2 or in *drm12cmt23* that lacks both H3K9me2 and CHH methylation (Fig. 6C). We found that both H3K9me2 and H3K27me1 are highly prevalent at C2 loci. Thus, our findings implicate a role of H3K27me1 in P4siRNA biogenesis at C2 loci (Fig. 7).

## Methods

### Plant materials

All tissues used in this study are from unopened flower buds and all *Arabidopsis* strains are in the Columbia ecotype. The *dcl2-1 dcl3-1 dcl4-2* (*dcl234*), *nrpd1-3* (*nrpd1*), and *rdr2-1* (*rdr2*) lines were previously described (Xie et al. 2004; Onodera et al. 2005; Henderson et al. 2006). The quadruple mutants *dcl234 nrpd1* and *dcl234 rdr2* were obtained by crossing of *dcl234* with *nrpd1* and *rdr2*.

### RNA isolation, digestion, and RT-PCR

Total RNAs were extracted from unopened flower buds with TRIzol (Invitrogen, 15596-018) and treated with DNase I (Roche, 04716728001). cDNA was synthesized using random primers with RevertAid Reverse Transcriptase (Fermentas EP0442). To determine the strandedness of the transcripts, reverse transcription was performed with gene-specific primers from each of the two strands. Sequences of primers are in Supplemental Table S4.

To determine the nature of the 5′ ends of P4RNAs, 5 μg total RNAs from *dcl234* were divided into each of four tubes and were treated as follows. First, the RNAs were incubated for 2 h at 37°C with or without enzymes: tube 1 and tube 2 with buffer only; tube 3 with tobacco acid pyrophosphatase (TAP; Epicentre T19250); and tube 4 with T4 Polynucleotide Kinase (PNK, NEB, M0201S). After phenol-chloroform extraction and ethanol precipitation, RNAs in tube 1 were incubated for 1 h at 30°C with buffer only, whereas RNAs in the other three tubes were incubated with Terminator Exonuclease (Epicentre, TER51020) for 1 h at 30°C. The RNAs were extracted with phenol-chloroform and precipitated with ethanol before being subjected to RT-PCR.

### Assembly of Pol IV/RDR2-dependent transcripts

In-house R scripts provided in the Supplemental Material under Supplemental Codes were used to assemble P4RNAs. The first step was to collect and combine all the reads located at P4RNA regions from the three replicates of dsRNA-seq libraries from *dcl234*. Then neighboring reads no more than 60 nt apart were joined together to form transcripts. The transcripts that passed the following three filters were retained. First, the transcripts must be longer than 60 nt. Second, the normalized read count from the combined three libraries of *dcl234* was >1 RPM. Third, the levels of the transcripts in *dcl234* were at least fourfold higher than those in *dcl234 nrpd1*. Finally, the transcripts were overlapped with P4siRNA loci to filter out the transcripts without corresponding P4siRNA expression.

### Determination of A/T composition of various genomic regions

Exons and genes were according to TAIR10 annotation; P4RNAs were determined in this study as described above. Only P4RNAs longer than 200 bp were included in this analysis. The start and end sites of P4RNA regions were arbitrarily defined as the 5′ and 3′ ends of P4RNAs on the Watson strand. Within each category (P4RNAs, exons, or genes), sequences were aligned at the start site of transcription (for P4RNAs and genes) or the beginning of exons, or at the end site of transcription/end of exons. Up to 1 kb of sequences flanking these sites were interrogated. The number of A, T, C, or G at each position for all the sequences in each category was counted. The A/T composition was calculated as the proportion of A and T nucleotides in the total. The Perl script is provided in the Supplemental Material under Supplemental Codes.

### Determination of nucleosome occupancy at various genomic regions

Nucleosome occupancy was examined at the same exons, genes, and P4RNAs interrogated for their A/T composition (described above). The positions of nucleosomes (Supplemental Dataset 2) in the genome were obtained by analysis of the data set (Chodavarapu et al. 2010) using the nucleosome-calling program NOrMAL (Polishko et al. 2012). The sequences of each category (P4RNAs, exons, or genes) were aligned at the start site of transcription (for P4RNAs and genes) or the beginning of exons, or at the end site of transcription/end of exons. For each position, the percentage of sequences with nucleosomes in total sequences was calculated. The Perl script is provided in the Supplemental Material under Supplemental Codes.

### A naïve method of identifying spliced reads

To identify reads that represent potential splicing events, the first step was to filter out reads that mapped perfectly to the genome. The unmapped reads were mapped to the genome again using blastall with a minimum mapped length of 15 nt (Zhang and Madden 1997). The reads were kept if both the beginning 15 nt and the end 15 nt of the reads mapped perfectly to the genome. In addition, the mapping positions on the genome of these reads were examined. If both the beginning and the end of the reads were mapped to the same strand within a distance of 1000 nt, the reads were kept as representing a potential splicing event. The scripts are provided in the Supplemental Material under Supplemental Codes.

### The definition of D2 and C2 loci

Differentially methylated regions (DMRs) in wild type versus *drm2* and wild type versus *cmt2*, named D2 and C2 DMRs, respectively, were derived from published methylome data sets (Stroud et al. 2013) with accession numbers listed in Supplemental Table S3. P4siRNA regions overlapping with D2 and C2 DMRs were referred to as D2 and C2 siRNA loci, respectively.

### The overlap between P4siRNAs with H3K27me1 and H3K9me2

The regions with H3K9me2 modifications were defined through analysis of published ChIP-chip data set (Roudier et al. 2011; Deleris et al. 2012) using BLOC (Pauler et al. 2009). The regions with H3K27me1 modifications were obtained in a published ChIP-chip data set (Roudier et al. 2011; Deleris et al. 2012). To calculate the P4siRNA regions with H3K27me1 and H3K9me2 modifications and the regions of H3K27me1 and H3K9me2 with P4siRNAs, the regions of H3K27me1 and H3K9me2 were divided into 500-bp arbitrary windows, and the overlap between these windows and those of P4RNAs was determined. Then the percentage of the overlap in total windows was determined.

### Supplemental methods

Detailed methods included in the Supplemental Material describe the construction of RNA-seq, RNA-seq-DSN, dsRNA-seq, and sRNA-seq libraries; the processing and mapping of RNA-seq, RNA-seq-DSN, dsRNA-seq; and sRNA-seq reads.

## Data access

The genome-wide data sets generated in this study are available at the NCBI Gene Expression Omnibus (GEO; http://www.ncbi.nlm.nih.gov/geo/) under accession number GSE57215.

## Supplementary Material

Supplemental Material

## References

[B1] Allen E, Xie Z, Gustafson AM, Carrington JC 2005 microRNA-directed phasing during *trans*-acting siRNA biogenesis in plants. Cell 121: 207–221. 15851028 10.1016/j.cell.2005.04.004

[B2] Ammar R, Torti D, Tsui K, Gebbia M, Durbic T, Bader GD, Giaever G, Nislow C 2012 Chromatin is an ancient innovation conserved between Archaea and Eukarya. eLife 1: e00078. 23240084 10.7554/eLife.00078 PMC3510453

[B3] Cao X, Jacobsen SE 2002 Locus-specific control of asymmetric and CpNpG methylation by the *DRM* and *CMT3* methyltransferase genes. Proc Natl Acad Sci (Suppl 4) 99: 16491–16498. 12151602 10.1073/pnas.162371599 PMC139913

[B4] Cho SH, Addo-Quaye C, Coruh C, Arif MA, Ma Z, Frank W, Axtell MJ 2008 *Physcomitrella patens DCL3* is required for 22–24 nt siRNA accumulation, suppression of retrotransposon-derived transcripts, and normal development. PLoS Genet 4: e1000314. 19096705 10.1371/journal.pgen.1000314 PMC2600652

[B5] Chodavarapu RK, Feng S, Bernatavichute YV, Chen PY, Stroud H, Yu Y, Hetzel JA, Kuo F, Kim J, Cokus SJ, 2010 Relationship between nucleosome positioning and DNA methylation. Nature 466: 388–392. 20512117 10.1038/nature09147 PMC2964354

[B6] Dahlber AE, Dahlber JE, Lund E, Tokimatsu H, Rabson AB, Calvert PC, Reynold SF, Zahalak M 1978 Processing of the 5′ end of *Escherichia coli* 16S ribosomal RNA. Proc Natl Acad Sci 75: 3598–3602. 358190 10.1073/pnas.75.8.3598 PMC392832

[B7] Deleris A, Stroud H, Bernatavichute Y, Johnson E, Klein G, Schubert D, Jacobsen SE 2012 Loss of the DNA methyltransferase MET1 Induces H3K9 hypermethylation at PcG target genes and redistribution of H3K27 trimethylation to transposons in *Arabidopsis thaliana*. PLoS Genet 8: e1003062. 23209430 10.1371/journal.pgen.1003062 PMC3510029

[B8] Erhard KF Jr, Stonaker JL, Parkinson SE, Lim JP, Hale CJ, Hollick JB 2009 RNA polymerase IV functions in paramutation in *Zea mays*. Science 323: 1201–1205. 19251626 10.1126/science.1164508

[B9] Haag JR, Ream TS, Marasco M, Nicora CD, Norbeck AD, Pasa-Tolic L, Pikaard CS 2012 In vitro transcription activities of Pol IV, Pol V, and RDR2 reveal coupling of Pol IV and RDR2 for dsRNA synthesis in plant RNA silencing. Mol Cell 48: 811–818. 23142082 10.1016/j.molcel.2012.09.027 PMC3532817

[B10] Henderson IR, Zhang X, Lu C, Johnson L, Meyers BC, Green PJ, Jacobsen SE 2006 Dissecting *Arabidopsis thaliana* DICER function in small RNA processing, gene silencing and DNA methylation patterning. Nat Genet 38: 721–725. 16699516 10.1038/ng1804

[B11] Herr AJ, Jensen MB, Dalmay T, Baulcombe DC 2005 RNA polymerase IV directs silencing of endogenous DNA. Science 308: 118–120. 15692015 10.1126/science.1106910

[B12] Jia Y, Lisch DR, Ohtsu K, Scanlon MJ, Nettleton D, Schnable PS 2009 Loss of RNA-dependent RNA polymerase 2 (RDR2) function causes widespread and unexpected changes in the expression of transposons, genes, and 24-nt small RNAs. PLoS Genet 5: e1000737. 19936292 10.1371/journal.pgen.1000737 PMC2774947

[B13] Johnson LM, Du J, Hale CJ, Bischof S, Feng S, Chodavarapu RK, Zhong X, Marson G, Pellegrini M, Segal DJ, 2014 SRA- and SET-domain-containing proteins link RNA polymerase V occupancy to DNA methylation. Nature 507: 124–128. 24463519 10.1038/nature12931 PMC3963826

[B14] Kim D, Pertea G, Trapnell C, Pimentel H, Kelley R, Salzberg SL 2013 TopHat2: accurate alignment of transcriptomes in the presence of insertions, deletions and gene fusions. Genome Biol 14: R36. 23618408 10.1186/gb-2013-14-4-r36 PMC4053844

[B15] Law JA, Jacobsen SE 2010 Establishing, maintaining and modifying DNA methylation patterns in plants and animals. Nat Rev Genet 11: 204–220. 20142834 10.1038/nrg2719 PMC3034103

[B16] Law JA, Ausin I, Johnson LM, Vashisht AA, Zhu JK, Wohlschlegel JA, Jacobsen SE 2010 A protein complex required for polymerase V transcripts and RNA-directed DNA methylation in *Arabidopsis*. Current Biol 20: 951–956. 10.1016/j.cub.2010.03.062 PMC2972704 20409711

[B17] Law JA, Vashisht AA, Wohlschlegel JA, Jacobsen SE 2011 SHH1, a homeodomain protein required for DNA methylation, as well as RDR2, RDM4, and chromatin remodeling factors, associate with RNA polymerase IV. PLoS Genet 7: e1002195. 21811420 10.1371/journal.pgen.1002195 PMC3141008

[B18] Law JA, Du J, Hale CJ, Feng S, Krajewski K, Palanca AM, Strahl BD, Patel DJ, Jacobsen SE 2013 Polymerase IV occupancy at RNA-directed DNA methylation sites requires SHH1. Nature 498: 385–389. 23636332 10.1038/nature12178 PMC4119789

[B19] Lee TF, Gurazada SG, Zhai J, Li S, Simon SA, Matzke MA, Chen X, Meyers BC 2012 RNA polymerase V-dependent small RNAs in Arabidopsis originate from small, intergenic loci including most SINE repeats. Epigenetics 7: 781–795. 22647529 10.4161/epi.20290 PMC3679228

[B20] Lister R, O’Malley RC, Tonti-Filippini J, Gregory BD, Berry CC, Millar AH, Ecker JR 2008 Highly integrated single-base resolution maps of the epigenome in *Arabidopsis*. Cell 133: 523–536. 18423832 10.1016/j.cell.2008.03.029 PMC2723732

[B21] Liu Q, Feng Y, Zhu Z 2009 Dicer-like (DCL) proteins in plants. Funct Integr Genomics 9: 277–286. 19221817 10.1007/s10142-009-0111-5

[B22] Matzke MA, Mosher RA 2014 RNA-directed DNA methylation: an epigenetic pathway of increasing complexity. Nat Rev Genet 15: 394–408. 24805120 10.1038/nrg3683

[B23] Mosher RA, Schwach F, Studholme D, Baulcombe DC 2008 PolIVb influences RNA-directed DNA methylation independently of its role in siRNA biogenesis. Proc Natl Acad Sci 105: 3145–3150. 18287047 10.1073/pnas.0709632105 PMC2268599

[B24] Onodera Y, Haag JR, Ream T, Costa Nunes P, Pontes O, Pikaard CS 2005 Plant nuclear RNA polymerase IV mediates siRNA and DNA methylation-dependent heterochromatin formation. Cell 120: 613–622. 15766525 10.1016/j.cell.2005.02.007

[B25] Pauler FM, Sloane MA, Huang R, Regha K, Koerner MV, Tamir I, Sommer A, Aszodi A, Jenuwein T, Barlow DP 2009 H3K27me3 forms BLOCs over silent genes and intergenic regions and specifies a histone banding pattern on a mouse autosomal chromosome. Genome Res 19: 221–233. 19047520 10.1101/gr.080861.108 PMC2652204

[B26] Polishko A, Ponts N, Le Roch KG, Lonardi S 2012 NORMAL: accurate nucleosome positioning using a modified Gaussian mixture model. Bioinformatics 28: i242–i249. 22689767 10.1093/bioinformatics/bts206 PMC3371838

[B27] Pontier D, Yahubyan G, Vega D, Bulski A, Saez-Vasquez J, Hakimi MA, Lerbs-Mache S, Colot V, Lagrange T 2005 Reinforcement of silencing at transposons and highly repeated sequences requires the concerted action of two distinct RNA polymerases IV in *Arabidopsis*. Genes Dev 19: 2030–2040. 16140984 10.1101/gad.348405 PMC1199573

[B28] Qi Y, He X, Wang XJ, Kohany O, Jurka J, Hannon GJ 2006 Distinct catalytic and non-catalytic roles of ARGONAUTE4 in RNA-directed DNA methylation. Nature 443: 1008–1012. 16998468 10.1038/nature05198

[B29] Roudier F, Ahmed I, Bérard C, Sarazin A, Mary-Huard T, Cortijo S, Bouyer D, Caillieux E, Duvernois-Berthet E, Al-Shikhley L, 2011 Integrative epigenomic mapping defines four main chromatin states in Arabidopsis. EMBO J 30: 1928–1938. 21487388 10.1038/emboj.2011.103 PMC3098477

[B30] Smith LM, Pontes O, Searle I, Yelina N, Yousafzai FK, Herr AJ, Pikaard CS, Baulcombe DC 2007 An SNF2 protein associated with nuclear RNA silencing and the spread of a silencing signal between cells in *Arabidopsis*. Plant Cell 19: 1507–1521. 17526749 10.1105/tpc.107.051540 PMC1913737

[B31] Stroud H, Greenberg MV, Feng S, Bernatavichute YV, Jacobsen SE 2013 Comprehensive analysis of silencing mutants reveals complex regulation of the *Arabidopsis* methylome. Cell 152: 352–364. 23313553 10.1016/j.cell.2012.10.054 PMC3597350

[B32] Stroud H, Do T, Du J, Zhong X, Feng S, Johnson L, Patel DJ, Jacobsen SE 2014 Non-CG methylation patterns shape the epigenetic landscape in *Arabidopsis*. Nat Struct Mol Biol 21: 64–72. 24336224 10.1038/nsmb.2735 PMC4103798

[B33] Unfried I, Gruendler P 1990 Nucleotide sequence of the 5.8S and 25S rRNA genes and of the internal transcribed spacers from *Arabidopsis thaliana*. Nucleic Acids Res 18: 4011. 2100998 10.1093/nar/18.13.4011 PMC331127

[B34] Wang XB, Jovel J, Udomporn P, Wang Y, Wu Q, Li WX, Gasciolli V, Vaucheret H, Ding SW 2011 The 21-nucleotide, but not 22-nucleotide, viral secondary small interfering RNAs direct potent antiviral defense by two cooperative argonautes in *Arabidopsis thaliana*. Plant Cell 23: 1625–1638. 21467580 10.1105/tpc.110.082305 PMC3101545

[B35] Wierzbicki AT, Haag JR, Pikaard CS 2008 Noncoding transcription by RNA polymerase Pol IVb/Pol V mediates transcriptional silencing of overlapping and adjacent genes. Cell 135: 635–648. 19013275 10.1016/j.cell.2008.09.035 PMC2602798

[B36] Wierzbicki AT, Ream TS, Haag JR, Pikaard CS 2009 RNA polymerase V transcription guides ARGONAUTE4 to chromatin. Nat Genet 41: 630–634. 19377477 10.1038/ng.365 PMC2674513

[B37] Wierzbicki AT, Cocklin R, Mayampurath A, Lister R, Rowley MJ, Gregory BD, Ecker JR, Tang H, Pikaard CS 2012 Spatial and functional relationships among Pol V-associated loci, Pol IV-dependent siRNAs, and cytosine methylation in the *Arabidopsis* epigenome. Genes Dev 26: 1825–1836. 22855789 10.1101/gad.197772.112 PMC3426761

[B38] Xie Z, Johansen LK, Gustafson AM, Kasschau KD, Lellis AD, Zilberman D, Jacobsen SE, Carrington JC 2004 Genetic and functional diversification of small RNA pathways in plants. PLoS Biol 2: E104. 15024409 10.1371/journal.pbio.0020104 PMC350667

[B39] Zemach A, Kim MY, Hsieh PH, Coleman-Derr D, Eshed-Williams L, Thao K, Harmer SL, Zilberman D 2013 The *Arabidopsis* nucleosome remodeler DDM1 allows DNA methyltransferases to access H1-containing heterochromatin. Cell 153: 193–205. 23540698 10.1016/j.cell.2013.02.033 PMC4035305

[B40] Zhang J, Madden TL 1997 PowerBLAST: a new network BLAST application for interactive or automated sequence analysis and annotation. Genome Res 7: 649–656. 9199938 10.1101/gr.7.6.649 PMC310664

[B41] Zhang CJ, Zhou JX, Liu J, Ma ZY, Zhang SW, Dou K, Huang HW, Cai T, Liu R, Zhu JK, 2013 The splicing machinery promotes RNA-directed DNA methylation and transcriptional silencing in *Arabidopsis*. EMBO J 32: 1128–1140. 23524848 10.1038/emboj.2013.49 PMC3630354

[B42] Zheng B, Wang Z, Li S, Yu B, Liu JY, Chen X 2009 Intergenic transcription by RNA polymerase II coordinates Pol IV and Pol V in siRNA-directed transcriptional gene silencing in *Arabidopsis*. Genes Dev 23: 2850–2860. 19948763 10.1101/gad.1868009 PMC2800093

[B43] Zheng Q, Ryvkin P, Li F, Dragomir I, Valladares O, Yang J, Cao K, Wang LS, Gregory BD 2010 Genome-wide double-stranded RNA sequencing reveals the functional significance of base-paired RNAs in *Arabidopsis*. PLoS Genet 6: e1001141. 20941385 10.1371/journal.pgen.1001141 PMC2947979

[B44] Zhong X, Hale CJ, Law JA, Johnson LM, Feng S, Tu A, Jacobsen SE 2012 DDR complex facilitates global association of RNA polymerase V to promoters and evolutionarily young transposons. Nat Struct Mol Biol 19: 870–875. 22864289 10.1038/nsmb.2354 PMC3443314

[B45] Zhong X, Du J, Hale CJ, Gallego-Bartolome J, Feng S, Vashisht AA, Chory J, Wohlschlegel JA, Patel DJ, Jacobsen SE 2014 Molecular mechanism of action of plant DRM de novo DNA methyltransferases. Cell 157: 1050–1060. 24855943 10.1016/j.cell.2014.03.056 PMC4123750

