# Supplementary material for: Detection of Pol IV/RDR2-dependent transcripts at the genomic scale in Arabidopsis reveals features and regulation of siRNA biogenesis
Source: Genome Res. 2015 Feb;25(2):235–45. doi: 10.1101/gr.182238.114 (PMC4315297; doi:10.1101/gr.182238.114)
Supplement: Supplemental Material [file supp_25_2_235__index.html]

Detection of Pol IV/RDR2-dependent transcripts at the genomic scale in Arabidopsis reveals features and regulation of siRNA biogenesis — Supplemental Material 

# Detection of Pol IV/RDR2-dependent transcripts at the genomic scale in *Arabidopsis* reveals features and regulation of siRNA biogenesis

## Supplemental Material

**Files in this Data Supplement:**

- Supp Codes.zip
- Supp Material.docx
- Supplemental Material.zip
