# Supplementary material for: Detection of Pol IV/RDR2-dependent transcripts at the genomic scale in Arabidopsis reveals features and regulation of siRNA biogenesis
Source: Genome Res. 2015 Feb;25(2):235–45. doi: 10.1101/gr.182238.114 (PMC4315297; doi:10.1101/gr.182238.114)
Supplement: Supplemental Material [file supp_gr.182238.114_SuppMaterial.docx]

**SUPPLEMENTAL MATERIAL**

Li et al.

**Summary**

Supplemental Methods

Supplemental Figures and Legends

Supplemental Tables

Supplemental References

Supplemental_Dataset_1 (separate document)

Supplemental_Dataset_2 (separate document)

Supplemental_Codes.zip (separate document)

**Supplemental Methods**

**Construction and sequencing of RNA-seq, RNA-seq-DSN, dsRNA-seq and sRNA-seq libraries**

Unopened flower buds from *dcl234* and *dcl234 nrpd1* were collected and were used for RNA extraction using Trizol (Invitrogen, 15596-018). Briefly, 10μg of DNA-free RNAs were subjected to rRNA removal using a Ribomius kit (Invitrogen, A10838-08). For dsRNA-seq libraries, RNase One (Promega, M4261) was used to digest single-stranded RNAs. The treated RNAs were fragmented using Fragmentation Reagents (Ambion, AM8740). T4 Polynucleotide Kinase (NEB, M0201S) was used to phosphorylate the 5’ ends as well as to remove the 3’ phosphate groups of the RNA fragments. The treated RNAs were resolved in a 15% denaturing polyacrylamide gel and 15-100 nt RNAs were excised and purified. These RNAs were used to construct the RNA-seq and dsRNA-seq libraries using the True-seq small RNA preparation kit (illumina, RS-200-0012). For some samples, the RNAs were further treated with Duplex-Specific Nuclease (DSN, Evrogen, EA001) to enrich for low abundance transcripts. The RNA-seq libraries treated with DSN are referred to as RNA-seq-DSN libraries. The sRNA-seq libraries were also constructed using the True-seq small RNA preparation kit. The libraries were sequenced through Illumina Hiseq2000 and the data were deposited at NCBI under the accession number GSE57215. All libraries built in this study are listed in Table S5, which contains information on the number of biological replicates for each library type and genotype.

**Processing and mapping of RNA-seq, RNA-seq-DSN and dsRNA-seq reads**

Raw reads were first collapsed into a set of non-redundant reads. All of the non-redundant reads were initially mapped to the *Arabidopsis* TAIR10 reference genome using the short-read alignment tool (BWA) allowing no mismatches (Henderson et al. 2006; Li and Durbin 2009).  Unaligned reads were processed further by sequentially trimming off nucleotides at the 3’ end with any match to the 5’ end of the adapter sequence allowing for 0, 1, 2, and 3 mismatches if the 3’ end nucleotides match to less than nine nucleotides, 10-19 nucleotides, 20-29 nucleotides, and 30-33 nucleotides, respectively, of the adapter sequence.  The longest allowed match to the adapter sequence is set arbitrarily at 33 nucleotides to maintain the shortest trimmed reads at 18 nucleotides.  Adapter-trimmed reads were mapped to the TAIR10 genome allowing no mismatches. All mapped reads (untrimmed and adaptor-trimmed) were combined for further downstream analysis.

To determine the regions that harbor P4RNAs, the genome was tiled into 500 bp bins and the reads whose 5’ ends fall within a bin were considered as belonging to this bin. The numbers of reads were counted for each bin for both *dcl234* and *dcl234 nrpd1* and compared between the two genotypes. The fold change and p-value were calculated using edgeR for dsRNA-seq and RNA-seq (Robinson et al. 2010). The Poisson distribution is used to calculate the p-value for RNA-seq-DSN libraries (Marioni et al. 2008). The regions with p-value < 0.01 and four-fold reduction in read counts in *dcl234 nrpd1* relative to *dcl234* were considered as regions that generate P4RNAs.

**Processing and mapping of sRNA-seq reads**

The reads in sRNA-seq libraries were first trimmed to remove adapters. Each read was queried for the presence of the first 9 nt sequence (TGGAATTCT) of the 5’ end adapter. If found, the query sequence plus the flanking 3’ end sequence is removed from the read. Adaptor-free reads between 18 nt and 42 nt in length were mapped to the TAIR10 genome. To calculate and compare small RNA abundance in different genotypes, the genome was tiled into 500 bp windows and reads whose 5’ end nucleotides fall within a window were assigned to the window. To identify differentially expressed small RNAs, edgeR was applied to calculate the fold change and p-value. The windows with p-value < 0.01 and four-fold reduction in read counts in *nrpd1* relative to WT were considered as regions that generate P4siRNAs.

**Supplemental Figures and Legends**

**Figure S1.** Genome-browser views of P4RNA and small RNA reads at two P4siRNA loci on chromosome 1. The two loci are the same as the ones shown in Figure 1A and 1B, except that three biological replicates (rep) are shown separately here. Note that reads from the two strands are not separately displayed.

**Figure S2.** Detection of P4RNAs. A, Random-primed RT-PCR to detect P4RNAs at 16 individual loci in *dcl234* and *dcl234 nrpd1*. Genomic DNA and H_2_O (no RNAs in the reactions) were included as positive and negative controls, respectively. -RT, reverse transcription was conducted in the absence of reverse transcriptase. B, The percentage of reads that map to genes, intergenic regions and P4siRNA loci in RNA-seq and dsRNA-seq. Three biological replicates (rep) are shown.

**Figure S3.** P4RNAs are derived from both DNA strands. RT-PCR was performed with random primers or strand-specific primers for reverse transcription (RT) and sequence-specific primers for PCR to detect P4RNAs. The nature of the RT primers is indicated below the gel images. The Watson strand refers to the reference strand in TAIR10 annotation; the Crick strand refers to the reverse complementary strand of the reference. -RT, reverse transcription was performed in the absence of reverse transcriptase.

**Figure S4.** Size distribution of P4RNAs. The number of P4RNAs in different size ranges (in nucleotide) is shown.

**Figure S5.** Relationships among P4RNAs, P4siRNAs, and CHH DNA methylation. A, The percentage of P4RNA regions that overlap with genes, transposons, repeats, and intergenic regions. B, The presence of P4siRNAs at loci dependent on DRM2 or CMT2 for CHH methylation. The percentage of DRM2- or CMT2-dependent loci with P4siRNAs is shown. DRM2- and CMT2-dependent loci were defined as differentially methylated CHH regions (DMRs) in *drm2* and *cmt2*, respectively, relative to wild type. C, Venn diagram showing the overlap among DMRs dependent on Pol IV, DRM2, or CMT2.

**Figure S6.** Chromosomal distributions of P4RNAs and other genomic features. A, The chromosomal distribution of annotated genes and genes overlapping with P4RNAs. B, The chromosomal distribution of P4RNAs and CHH methylated regions. C, The chromosomal distribution of P4RNAs and regions containing H3K27me1 or H3K9me2. In A-C, the outermost layer represents each of the five chromosomes, with the centromeres indicated by the black bands. The inner layers represent the density of the featured regions (color coded) in 5 kb windows.

**Figure S7.** Features of P4RNAs and Pol II transcribed RNAs at P4siRNA loci. A, A genome-browser view of reads from sRNA-seq and polyA- RNA-seq at a P4siRNA locus on chromosome 1. This locus is also shown in Figure 3E; two biological replicates (rep) are shown here. B, A genome-browser view of reads from sRNA-seq and polyA+ RNA-seq at a P4siRNA locus on chromosome 2. This locus is also shown in Figure 3F; two biological replicates are shown here. Normalized read numbers are shown above or below the horizontal lines for reads from the Watson and Crick strands, respectively. C, The percentage of transcripts derived from one major strand at P4siRNA loci in polyA+ RNA-seq. The numbers of reads from each of the two strands at P4siRNA loci were counted in polyA+ RNA-seq. Loci with 90% of the reads derived from one strand were considered as loci with transcripts derived from one major strand.

**Figure S8.** Plots showing the strandedness of small RNAs and polyA+ RNAs from P4siRNA loci with Pol II transcribed RNAs. The x-axis and y-axis represent the numbers of raw reads from the Watson and Crick strands, respectively. Each dot represents one P4siRNA locus, with the green and red colors representing small RNAs and polyA+ RNAs, respectively. Results from each of two biological replicates (rep) of polyA+ RNA-seq and the corresponding sRNA-seq from *dcl234* and *dcl234 nrpd1* are shown as indicated.

**Figure S9.** The presence of P4RNAs in *dcl234* is correlated with the levels of CHH methylation but not P4siRNA abundance. A, A pie chart showing the reasons why P4RNAs were not detected by comparing *dcl234* to *dcl234 nrpd1* in dsRNA-seq. Low read abundance was defined as a total read count of less than 0.9RPM in all three *dcl234* libraries at a particular P4siRNA locus. The loci with p-value > 0.01 showed a consistent reduction in read abundance in *dcl234 nrpd1* but did not pass the p-value filter for the annotation of P4RNAs. B, The relative abundance of D2 and C2 siRNAs as determined by two replicates of sRNA-seq. P4siRNAs of 21nt, 22nt, 23nt, 24nt and 18-42nt (total) are shown. C and D, A lack of correlation between the ability to detect P4RNAs and the abundance of siRNAs at the corresponding loci in WT. P4siRNA loci were divided into four quartiles according to P4siRNA abundance in WT with the first quartile being loci containing the most abundant P4siRNAs. C, The CHH methylation level in WT for the four quartiles of loci. siRNA loci with or without P4RNA detected in our dsRNA-seq (comparing *dcl234* and *dcl234 nrpd1*) are shown separately; the two types of loci do not show drastic differences in their levels of CHH methylation in WT. D, The CHH methylation level for the four quartiles of loci in *dcl234*. P4siRNA loci without P4RNAs detected in our dsRNA-seq have lower CHH methylation in *dcl234* relative to loci with P4RNAs detected.

**Figure S10.** Differences between D2 and C2 loci in P4RNA discovery, P4siRNA levels, and CHH methylation levels. A-C, D2 and C2 siRNA loci were divided into four quartiles according to their CHH methylation levels in *dcl234*. A, The relative abundance of P4RNAs in *dcl234* in each quartile. B, Average CHH methylation levels in *dcl234* in the four quartiles. C, The relative abundance of P4siRNAs in *dcl234* in each quartile. D-E, D2 and C2 loci were divided into four quartiles according to their CHH methylation levels in WT. D, The average CHH methylation levels in WT in the four quartiles. E, The relative abundance of P4siRNAs in WT in each quartile.

**Figure S11.** CHH methylation, H3K27me1, and H3K9me2 levels in WT and various mutants. A-B, CHH methylation levels in various genotypes at D2 (A) and C2 (B) loci. CHH methylation levels were determined (see Methods) using published methylome data (Stroud et al. 2013; Stroud et al. 2014). C-D, H3K27me1 levels at D2 and C2 loci as determined by ChIP-chip (Roudier et al. 2011). C, The number of probes that show H3K27me1 signals at various genomic features in the published ChIP-chip study. D, The average H3K27me1 ChIP-chip signal intensity at the indicated genomic regions corresponding to the probes in C. Results from two biological replicates (rep) are shown separately. E-F, H3K9me2 levels at D2 and C2 loci as determined by ChIP-chip (Deleris et al. 2012). E, The number of probes with H3K9me2 signals at various genomic features in the published ChIP-chip study. F, The average H3K9me2 signal intensity at the indicated genomic regions corresponding to the probes in E. Results from two biological replicates (rep) are shown separately.

| **Supplemental Tables**  Table S1. Chromosomal positions of the P4siRNA loci examined by RT-PCR in this study | | | |
| --- | --- | --- | --- |
| Name | Chromosome | Start Position | End Position |
| Locus 1 | Chr1 | 11619088 | 11619830 |
| Locus 2 | Chr3 | 5780028 | 5780762 |
| Locus 3 | Chr3 | 7419920 | 7421330 |
| Locus 4 | Chr3 | 10691074 | 10691841 |
| Locus 5 | Chr3 | 10747222 | 10748309 |
| Locus 6 | Chr1 | 4506452 | 4507032 |
| Locus 8 | Chr2 | 5661047 | 5661660 |
| Locus 13 | Chr2 | 2865442 | 2866452 |
| Locus 20 | Chr3 | 11042663 | 11043163 |
| Locus 23 | Chr3 | 14729148 | 14731788 |
| Locus 26 | Chr3 | 15682149 | 15682550 |
| Locus 27 | Chr3 | 17842320 | 17843219 |
| Locus 28 | Chr3 | 20030863 | 20031378 |
| Locus 30 | Chr1 | 23453816 | 23455008 |
| Locus 33 | Chr4 | 12841422 | 12842570 |
| Locus 38 | Chr4 | 272801 | 273244 |
| Locus 40 | Chr5 | 9800868 | 9801476 |
| Locus 41 | Chr5 | 1410300 | 1410550 |
| Locus 42 | Chr5 | 17174556 | 17175364 |
| Locus 43 | Chr5 | 20313464 | 20314875 |
| Locus 44 | Chr5 | 22706840 | 22707252 |
| Locus 45 | Chr5 | 22707688 | 22708114 |

Table S2. GO annotation of genes overlapping with P4RNAs

| GO ID | Gene^1^ number | Gene^2^ number | p-value | Adjusted p-value | GO Term | GO category |
| --- | --- | --- | --- | --- | --- | --- |
| GO:0004565 | 40 | 12 | 9.37E-10 | 7.59E-08 | beta-galactosidase activity | molecular function |
| GO:0015925 | 44 | 12 | 3.18E-09 | 2.57E-07 | galactosidase activity | molecular function |
| GO:0016798 | 442 | 33 | 1.02E-06 | 8.25E-05 | hydrolase activity, acting on glycosyl bonds | molecular function |
| GO:0005199 | 39 | 9 | 1.43E-06 | 1.16E-04 | structural constituent of cell wall | molecular function |
| GO:0004553 | 412 | 31 | 1.83E-06 | 1.48E-04 | hydrolase activity, hydrolyzing O-glycosyl compounds | molecular function |
| GO:0030145 | 39 | 8 | 1.41E-05 | 1.14E-03 | manganese ion binding | molecular function |
| GO:0030599 | 147 | 15 | 2.80E-05 | 2.27E-03 | pectinesterase activity | molecular function |
| GO:0004650 | 71 | 10 | 3.94E-05 | 3.19E-03 | polygalacturonase activity | molecular function |
| GO:0007047 | 165 | 16 | 3.50E-05 | 1.58E-03 | cell wall organization | biological process |
| GO:0045229 | 183 | 16 | 1.21E-04 | 5.45E-03 | external encapsulating structure organization | biological process |
| GO:0070882 | 229 | 18 | 1.82E-04 | 8.18E-03 | cell wall organization or biogenesis | biological process |
| GO:0012505 | 4063 | 207 | 2.10E-15 | 4.62E-14 | endomembrane system | cellular component |
| GO:0009341 | 30 | 10 | 9.42E-09 | 2.07E-07 | beta-galactosidase complex | cellular component |

1 All annotated genes

2 Genes overlapping with P4RNAs

Table S3. Published genomic datasets used in this study

| Library | Genotype | Geo ID | Publication |
| --- | --- | --- | --- |
| ChIP-chip (H3K9me2) | Col | [GSE37075](http://www.ncbi.nlm.nih.gov/geo/query/acc.cgi?acc=GSE37075) | Deleris et al. 2012 |
| ChIP-chip  (H3K27me1) | Col | [GSE24710](http://www.ncbi.nlm.nih.gov/geo/query/acc.cgi?acc=GSE24710) | Roudier et al. 2011 |
| BS-seq | Col rep1 | [GSM938370](http://www.ncbi.nlm.nih.gov/geo/query/acc.cgi?acc=GSM938370) | Stroud et al. 2013 |
| BS-seq | Col rep2 | [GSM980986](http://www.ncbi.nlm.nih.gov/geo/query/acc.cgi?acc=GSM980986) | Stroud et al. 2013 |
| BS-seq | Col rep3 | [GSM980987](http://www.ncbi.nlm.nih.gov/geo/query/acc.cgi?acc=GSM980987) | Stroud et al. 2013 |
| BS-seq | *clsy1* | [GSM981000](http://www.ncbi.nlm.nih.gov/geo/query/acc.cgi?acc=GSM981000) | Stroud et al. 2013 |
| BS-seq | *cmt2* | [GSM981002](http://www.ncbi.nlm.nih.gov/geo/query/acc.cgi?acc=GSM981002) | Stroud et al. 2013 |
| BS-seq | *dcl234* | [GSM981008](http://www.ncbi.nlm.nih.gov/geo/query/acc.cgi?acc=GSM981008) | Stroud et al. 2013 |
| BS-seq | *dms3* | [GSM981010](http://www.ncbi.nlm.nih.gov/geo/query/acc.cgi?acc=GSM981010) | Stroud et al. 2013 |
| BS-seq | *dms4* | [GSM981011](http://www.ncbi.nlm.nih.gov/geo/query/acc.cgi?acc=GSM981011) | Stroud et al. 2013 |
| BS-seq | *drd1* | [GSM981014](http://www.ncbi.nlm.nih.gov/geo/query/acc.cgi?acc=GSM981014) | Stroud et al. 2013 |
| BS-seq | *drm12* | [GSM981015](http://www.ncbi.nlm.nih.gov/geo/query/acc.cgi?acc=GSM981015) | Stroud et al. 2013 |
| BS-seq | *nrpd1* | [GSM981039](http://www.ncbi.nlm.nih.gov/geo/query/acc.cgi?acc=GSM981039) | Stroud et al. 2013 |
| BS-seq | *nrpe1* | [GSM981040](http://www.ncbi.nlm.nih.gov/geo/query/acc.cgi?acc=GSM981040) | Stroud et al. 2013 |
| BS-seq | *rdr2* | [GSM981044](http://www.ncbi.nlm.nih.gov/geo/query/acc.cgi?acc=GSM981044) | Stroud et al. 2013 |
| BS-seq | *rdm1* | [GSM981042](http://www.ncbi.nlm.nih.gov/geo/query/acc.cgi?acc=GSM981042) | Stroud et al. 2013 |
| BS-seq | *suvh456* | [GSM981060](http://www.ncbi.nlm.nih.gov/geo/query/acc.cgi?acc=GSM981060) | Stroud et al. 2013 |
| BS-seq | Col | [GSM1242401](http://www.ncbi.nlm.nih.gov/geo/query/acc.cgi?acc=GSM1242401) | Stroud et al. 2014 |
| BS-seq | *drm12cmt23* | [GSM1242404](http://www.ncbi.nlm.nih.gov/geo/query/acc.cgi?acc=GSM1242404) | Stroud et al. 2014 |
| sRNA-seq | Col | [GSM1242406](http://www.ncbi.nlm.nih.gov/geo/query/acc.cgi?acc=GSM1242406) | Stroud et al. 2014 |
| sRNA-seq | *cmt2* | [GSM1242407](http://www.ncbi.nlm.nih.gov/geo/query/acc.cgi?acc=GSM1242407) | Stroud et al. 2014 |
| sRNA-seq | *drm12cmt23* | [GSM1242409](http://www.ncbi.nlm.nih.gov/geo/query/acc.cgi?acc=GSM1242409) | Stroud et al. 2014 |
| sRNA-seq | *suvh456* | [GSM1242410](http://www.ncbi.nlm.nih.gov/geo/query/acc.cgi?acc=GSM1242410) | Stroud et al. 2014 |
| sRNA-seq | Col | [GSM893118](http://www.ncbi.nlm.nih.gov/geo/query/acc.cgi?acc=GSM893118) | Lee et al. 2012 |
| sRNA-seq | *dms4* | [GSM893119](http://www.ncbi.nlm.nih.gov/geo/query/acc.cgi?acc=GSM893119) | Lee et al. 2012 |
| sRNA-seq | *drd1* | [GSM893120](http://www.ncbi.nlm.nih.gov/geo/query/acc.cgi?acc=GSM893120) | Lee et al. 2012 |
| sRNA-seq | *dms3* | [GSM893121](http://www.ncbi.nlm.nih.gov/geo/query/acc.cgi?acc=GSM893121) | Lee et al. 2012 |
| sRNA-seq | *rdm1* | [GSM893122](http://www.ncbi.nlm.nih.gov/geo/query/acc.cgi?acc=GSM893122) | Lee et al. 2012 |
| sRNA-seq | Col rep1 | [GSM1103235](http://www.ncbi.nlm.nih.gov/geo/query/acc.cgi?acc=GSM1103235) | Law et al. 2013 |
| sRNA-seq | Col rep2 | [GSM1103236](http://www.ncbi.nlm.nih.gov/geo/query/acc.cgi?acc=GSM1103236) | Law et al. 2013 |
| sRNA-seq | *nrpe1* | [GSM1103238](http://www.ncbi.nlm.nih.gov/geo/query/acc.cgi?acc=GSM1103238) | Law et al. 2013 |
| sRNA-seq | *drm2* | [GSM1103240](http://www.ncbi.nlm.nih.gov/geo/query/acc.cgi?acc=GSM1103240) | Law et al. 2013 |

| Table S4. Primers used in this study | |  |
| --- | --- | --- |
|  |  |  |
| Name | Sequence | Purpose |
| Locus 1F | AATACAAGCAACATAGGGAAG | RT-PCR for locus 1 Watson strand primer for RT |
| Locus 1R | AACCAAGCCACAAATCTCT | RT-PCR for locus 1 Crick strand primer for RT |
| Locus 2F | TATCGTATTGTCGTCCTTGA | RT-PCR for locus 2 Watson strand primer for RT |
| Locus 2R | GTCCCACTCCACTTTCATT | RT-PCR for locus 2 Crick strand primer for RT |
| Locus 3F | GGGAAACGACTTTGTATGTT | RT-PCR for locus 3 Watson strand primer for RT |
| Locus 3R | ATTGCTCTGGTGTTCTCACT | RT-PCR for locus 3 Crick strand primer for RT |
| Locus 4F | AGCATCCCCAATAACAAAT | RT-PCR for locus 4 Watson strand primer for RT |
| Locus 4R | ATCTACGAGGTCAGTCAAGG | RT-PCR for locus 4 Crick strand primer for RT |
| Locus 5F | CGAACAGCACCACTAAGC | RT-PCR for locus 5 Watson strand primer for RT |
| Locus 5R | GAAGGAAAAGCAACTCACTC | RT-PCR for locus 5 Crick strand primer for RT |
| Locus 6F | GCATCATTCACAGTATCCAA | RT-PCR for locus 6 Watson strand primer for RT |
| Locus 6R | GTTCTTCTTCTTCGGGTATC | RT-PCR for locus 6 Crick strand primer for RT |
| Locus 8F | AAAGAGATGTTGGTGAAAGG | RT-PCR for locus 8 |
| Locus 8R | CTTGATGGGTGGAATGAC | RT-PCR for locus 8 |
| Locus 13F | TAAGATTGATGTAACTGGGAAG | RT-PCR for locus 13 Watson strand primer for RT |
| Locus 13R | TCGGTAGAGATGACTTGAGA | RT-PCR for locus 13 Crick strand primer for RT |
| Locus 20F | GAACAAGGCTACTGTGGTG | RT-PCR for locus 20 Watson strand primer for RT |
| Locus 20R | GGAAGGCATCCATTTGAT | RT-PCR for locus 20 Crick strand primer for RT |
| Locus 23F | AAGAAAGCCCAAGTAGAAGA | RT-PCR for locus 23 |
| Locus 23R | AGCGTATCAACCCAAATG | RT-PCR for locus 23 |
| Locus 26F | AACTACCCCAATCCTTTCTA | RT-PCR for locus 26 |
| Locus 26R | CTGGTCACTTCTCCGATG | RT-PCR for locus 26 |

| Locus 27F | TACTCTTGGCTTCTCAAAAC | RT-PCR for locus 27 |
| --- | --- | --- |
| Locus 27R | CATTGTGTCCTCCTGTTACC | RT-PCR for locus 27 |
| Locus 28F | TGGATACTTGCCTCGTGT | RT-PCR for locus 28 |
| Locus 28R | CCAGATGGAGACATTATTG | RT-PCR for locus 28 |
| Locus 30F | ATAGCCTTCAACACTTGCTT | RT-PCR for locus 30 Watson strand primer for RT |
| Locus 30R | GAGTTCATTCTCCGACTTTC | RT-PCR for locus 30 Crick strand primer for RT |
| Locus 33F | CCAGAAGAATAGCATAGAAGC | RT-PCR for locus 33 |
| Locus 33R | TAGGAATACAAGACCTCAAATG | RT-PCR for locus 33 |
| Locus 38F | GATGGACTCTCTGGCTTG | RT-PCR for locus 38 |
| Locus 38R | AACGGTGGTGATTATGGA | RT-PCR for locus 38 |
| Locus 40F | ATTATTCAAACTCACCACAAAG | RT-PCR for locus 40 |
| Locus 40R | AATCGCCTTCACAACATTA | RT-PCR for locus 40 |
| Locus 41F | TGCTTTTCCTTCACTCTTCT | RT-PCR for locus 41 |
| Locus 41R | TAACGGCTCTATCACTTTTG | RT-PCR for locus 41 |
| Locus 42F | AGGGAGTAATAGATGTGATGG | RT-PCR for locus 42 |
| Locus 42R | ATTTAGGAGGAGCAAAAGC | RT-PCR for locus 42 |
| Locus 43F | GGTGTTGGATAAAGGGTAGA | RT-PCR for locus 43 |
| Locus 43R | CATCTTGTGAGCAGGAAAA | RT-PCR for locus 43 |
| Locus 44F | GTAAATAAACCCAAGAACCAC | RT-PCR for locus 44 |
| Locus 44R | TGCGAAACTAATGGAAGAAT | RT-PCR for locus 44 |
| Locus 45F | TTTGGTAGAATAGAAGGAATGA | RT-PCR for locus 45 |
| Locus 45R | TGAAATAAGATGGGGACAAT | RT-PCR for locus 45 |
| UBC-F | TACAGCGAGAGAAAGTAGCA | RT-PCR for *UBC21* |
| UBC-R | GCAAAGGATAAGGTTCAGG | RT-PCR for *UBC21* |
| CBP20-F | TCAGGAACACAAGAGGAGTT | RT-PCR for *CBP20* |
| CBP20-R | AGAACAGGACGAAACAAAAG | RT-PCR for *CBP20* |

| Tables S5. Genomic datasets generated in this study^1^ | |
| --- | --- |
|  |  |
| Library | Genotype |
| dsRNA-seq | *dcl2-1 dcl3-1 dcl4-2* rep1^2^ |
| dsRNA-seq | *dcl2-1 dcl3-1 dcl4-2* rep2 |
| dsRNA-seq | *dcl2-1 dcl3-1 dcl4-2* rep3 |
| dsRNA-seq | *dcl2-1 dcl3-1 dcl4-2 nrpd1-3* rep1 |
| dsRNA-seq | *dcl2-1 dcl3-1 dcl4-2 nrpd1-3* rep2 |
| dsRNA-seq | *dcl2-1 dcl3-1 dcl4-2 nrpd1-3* rep3 |
| RNA-seq | *dcl2-1 dcl3-1 dcl4-2* rep1 |
| RNA-seq | *dcl2-1 dcl3-1 dcl4-2* rep2 |
| RNA-seq | *dcl2-1 dcl3-1 dcl4-2* rep3 |
| RNA-seq | *dcl2-1 dcl3-1 dcl4-2 nrpd1-3* rep1 |
| RNA-seq | *dcl2-1 dcl3-1 dcl4-2 nrpd1-3* rep2 |
| RNA-seq | *dcl2-1 dcl3-1 dcl4-2 nrpd1-3* rep3 |
| RNA-seq-DSN | *dcl2-1 dcl3-1 dcl4-2* |
| RNA-seq-DSN | *dcl2-1 dcl3-1 dcl4-2 nrpd1-3* |
| RNA-seq-DSN | *dcl2-1 dcl3-1 dcl4-2* *rdr2-1* |
| RNA-seq (poly A+) | *dcl2-1 dcl3-1 dcl4-2* rep1 |
| RNA-seq (poly A+) | *dcl2-1 dcl3-1 dcl4-2* rep2 |
| RNA-seq (poly A+) | *dcl2-1 dcl3-1 dcl4-2 nrpd1-3* rep1 |
| RNA-seq (poly A+) | *dcl2-1 dcl3-1 dcl4-2 nrpd1-3* rep2 |
| RNA-seq (poly A-) | *dcl2-1 dcl3-1 dcl4-2* rep1 |
| RNA-seq (poly A-) | *dcl2-1 dcl3-1 dcl4-2* rep2 |
| RNA-seq (poly A-) | *dcl2-1 dcl3-1 dcl4-2 nrpd1-3* rep1 |
| RNA-seq (poly A-) | *dcl2-1 dcl3-1 dcl4-2 nrpd1-3* rep2 |
| sRNA-seq | Col rep1 |
| sRNA-seq | Col rep2 |
| sRNA-seq | *nrpd1-3* rep1 |
| sRNA-seq | *nrpd1-3* rep2 |
| sRNA-seq | *dcl2-1 dcl3-1 dcl4-2* rep1 |
| sRNA-seq | *dcl2-1 dcl3-1 dcl4-2* rep2 |
| sRNA-seq | *dcl2-1 dcl3-1 dcl4-2 nrpd1-3*  rep1 |
| sRNA-seq | *dcl2-1 dcl3-1 dcl4-2 nrpd1-3* rep2 |
| sRNA-seq | *rdr2-1* rep1 |
| sRNA-seq | *rdr2-2* rep2 |
| sRNA-seq | *dcl3-1* rep1 |
| sRNA-seq | *dcl3-1* rep2 |
| sRNA-seq | *clsy1* |
| 1 The datasets have been deposited in the Gene Expression Omnibus at National Center for Biotechnology Information under the accession number GSE57215.  2 rep: biological replicate | |

**Supplemental References**

Deleris A, Stroud H, Bernatavichute Y, Johnson E, Klein G, Schubert D, Jacobsen SE. 2012. Loss of the DNA methyltransferase MET1 Induces H3K9 hypermethylation at PcG target genes and redistribution of H3K27 trimethylation to transposons in Arabidopsis thaliana. *PLoS genetics* **8**(11): e1003062.

Marioni JC, Mason CE, Mane SM, Stephens M, Gilad Y. 2008. RNA-seq: an assessment of technical reproducibility and comparison with gene expression arrays. *Genome research* **18**(9): 1509-1517.

Robinson MD, McCarthy DJ, Smyth GK. 2010. edgeR: a Bioconductor package for differential expression analysis of digital gene expression data. *Bioinformatics* **26**(1): 139-140.

Roudier F, Ahmed I, Berard C, Sarazin A, Mary-Huard T, Cortijo S, Bouyer D, Caillieux E, Duvernois-Berthet E, Al-Shikhley L et al. 2011. Integrative epigenomic mapping defines four main chromatin states in Arabidopsis. *The EMBO journal* **30**(10): 1928-1938.

Stroud H, Do T, Du J, Zhong X, Feng S, Johnson L, Patel DJ, Jacobsen SE. 2014. Non-CG methylation patterns shape the epigenetic landscape in Arabidopsis. *Nature structural & molecular biology* **21**(1): 64-72.

Stroud H, Greenberg MV, Feng S, Bernatavichute YV, Jacobsen SE. 2013. Comprehensive analysis of silencing mutants reveals complex regulation of the Arabidopsis methylome. *Cell* **152**(1-2): 352-364.
